# Cinnamic‐Hydroxamic‐Acid Derivatives Exhibit Antibiotic, Anti‐Biofilm, and Supercoiling Relaxation Properties by Targeting Bacterial Nucleoid‐Associated Protein HU

**DOI:** 10.1002/advs.202509876

**Published:** 2025-11-21

**Authors:** Huan Chen, Yingqi Xu, Zhaoping Xiong, Hongliang Wang, Xin Wang, Yue Kang, Zhonglin Wang, Xiaoyan Zeng, Yahui Liu, Yehuan Zheng, Wei Chen, Mengzhe Li, Zhenyue Hu, Chi Xu, Yue Wu, Yawen Wang, Zuyi Yuan, Shuai Yuan, Huadong Liu, Steve Matthews, Nan Qiao, Yan Li, Bing Liu

**Affiliations:** ^1^ Department of Infectious Diseases The First Affiliated Hospital of Xi'an Jiaotong University Xi'an Jiaotong University 1st Affiliated Hospital Xi'an Shaanxi 710041 China; ^2^ Key Laboratory of Surgical Critical Care and Life Support BioBank Shaanxi Engineering Research Center for Biobank and Advanced Medical Research Department of Cardiology Department of Laboratory Medicine The First Affiliated Hospital of Xi'an Jiaotong University Xi'an Jiaotong University Xi'an 710041 China; ^3^ Department of Life Sciences Faculty of Natural Sciences Imperial College London Exhibition Road London SW7 2AZ United Kingdom; ^4^ Laboratory of Health Intelligence HW Cloud Computing Technologies Co., Ltd Guizhou China; ^5^ Key Laboratory of Tropical Translational Medicine of Ministry of Education & NHC Key Laboratory of Tropical Disease Control Hainan Academy of Medical Sciences Hainan Medical College Haikou China; ^6^ Department of Pathogen Biology School of Basic Medicine State Key Laboratory for Diagnosis and Treatment of Severe Zoonotic Infectious Diseases Tongji Medical College Huazhong University of Science and Technology Wuhan 430030 China; ^7^ College of Life Science and Technology Beijing University of Chemical Technology Beijing 100089 China; ^8^ Key Laboratory of Virology and Biosafety Wuhan Institute of Virology Chinese Academy of Sciences Wuhan 430071 China; ^9^ School of Life Science and health University of Health and Rehabilitation Sciences Qingdao 266113 China

**Keywords:** bacterial antibiotic resistance, biofilm, cinnamic‐hydroxamic‐acid derivatives, DNA supercoiling, HU

## Abstract

Finding novel compounds and drug targets is crucial for antibiotic development. The nucleoid‐associated protein HU plays a significant role in bacterial DNA metabolism, supercoiling, and biofilm formation, making it a promising new target. In this work, structure‐based screening and identified cinnamic‐hydroxamic‐acid derivatives (CHADs) are conducted as HU inhibitors, with a minimum inhibitory concentration (MIC) of as low as **12 µg mL^−1^** against a range of pathogenic bacteria. CHADs induce nucleoid deformation, preventing bacterial division and inhibiting growth. They exhibit low toxicity in mice and effectively treat infections in mouse models. Additionally, CHADs possess anti‐biofilm activity and supercoiling relaxation properties, countering bacterial stress responses to antibiotics. They suppress changes in gene expression required for optimal stress responses, resulting in synergistic effects with other antibiotics. Thus, CHADs represent a new class of antibiotics that inhibit bacterial stress responses by co‐targeting biofilm formation and DNA supercoiling.

## Graphical abstract

Cinnamic‐hydroxamic‐acid derivatives (CHADs) are identified as novel inhibitors of the bacterial nucleoid‐associated protein HU, exhibiting potent antibacterial, anti‐biofilm (both inhibition and eradication), and DNA relaxation (anti‐supercoiling) activities. Moreover, CHADs demonstrate strong synergistic effects with multiple antibiotics.

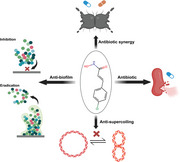

## 1 Introduction

Antimicrobial resistance (AMR) is a major threat to human health globally.^[^ ^1^ ^]^ The rise of AMR has been attributed to the overuse of existing antibiotics and the slow commercialization of new drugs.^[^ ^2^, ^3^, ^4^, ^5^ ^]^ Antibiotics combat bacteria through three main mechanisms: disrupting the synthesis of nucleic acids, the cell wall, or proteins by targeting key enzymes in these pathways.^[^ ^6^ ^]^ To combat AMR, extensive efforts have been made to identify new drug targets and develop novel therapeutic strategies.^[^ ^7^, ^8^, ^9^ ^]^ Among newly identified targets, HU, a nucleoid‐associated protein (NAP), plays critical roles in bacterial survival, growth, SOS response, adaptive mutation, cell division, biofilm formation, and many other cell processes.^[^ ^10^, ^11^ ^]^ HU is encoded by the *hup* gene(s) and is essential for many bacteria,^[^ ^12^ ^]^ while *hup* deletion in other bacteria, including *Escherichia coli* (*E. coli*) causes major, growth defects.^[^ ^13^ ^]^ Small‐molecule HU inhibitors have been developed and shown to inhibit the growth of various microbes. Targeting either the “pit” or the α‐helical region of the HUs, *trans*‐stilbene derivatives (SDs) and bisphenol derivatives of fluorene (BDFs) have been developed to intervene in the DNA binding properties of their target HUs. Based on the docked models, SDs have been suggested to break up the interaction between the basic residues in the “pit” and DNA in *Mycobacterium tuberculosis* (*M. tuberculosis*)^[^ ^14^ ^]^ and the African swine fever virus.^[^ ^15^ ^]^ In contrast, BDFs bind to the α‐helical region and interfere with the DNA binding “arm” and “pit” allosterically, and this class of compounds is effective against *Spiroplasma melliferum* (*S. melliferum*).^[^ ^16^ ^]^ Interestingly, Bacillus phage SPO1 is also known to produce a cross‐species HU inhibitor, Gp46.^[^ ^17^ ^]^ Although the inhibition mechanism of Gp46 differs from those of SDs and BDFs, as Gp46 does not bind HU via the proposed “pit” or α‐helical region, the existence of a phage HU inhibitor suggests that inhibiting HU would also facilitate phage‐mediated bacterial killing.

Notably, HU is not only indispensable for cell viability in many bacteria, but also plays critical roles in biofilm formation and the regulation of DNA supercoiling, both of which are crucial for bacterial stress responses to antibiotics.^[^ ^18^, ^19^, ^20^, ^21^, ^22^ ^]^ Biofilms comprise the bacterial cells embedded within a complex matrix of extracellular polymeric substances (EPS), which consists of polysaccharides, lipids, extracellular DNA (eDNA), and proteins.^[^ ^23^ ^]^ They provide a protective environment that significantly enhances bacterial survival against antibiotics and the host immune system.^[^ ^24^ ^]^ Importantly, HU has been shown to play a crucial role in biofilm formation by binding to eDNA and lipopolysaccharide (LPS), acting as a molecular glue.^[^ ^19^ ^]^ Notably, the eDNA‐based biofilm architecture relies on the rare Z‐DNA conformation, which is stabilized by DNABII family proteins such as HU and integration host factor (IHF).^[^ ^25^ ^]^ During biofilm development, Z‐DNA accumulates and is maintained by these proteins as the biofilm matures.^[^ ^26^, ^27^ ^]^ Meanwhile, the bacterial supercoiling results from the combined effects of transcription, replication, topoisomerase activity, and NAPs, including HU.^[^ ^28^ ^]^ HU can induce and constrain supercoiling by bending and coiling DNA,^[^ ^29^, ^30^, ^31^ ^]^ and its deletion reduces the supercoiling of both plasmid and chromosomal DNA.^[^ ^32^ ^]^ HU‐DNA interaction, likely through its role in adjusting DNA supercoiling, is considered as a general mechanism for bacterial transcriptional regulation during the cell cycle and stress responding, including antibiotics.^[^ ^20^, ^33^ ^]^ Since both biofilm formation and supercoiling are important for bacterial antibiotic resistance, a HU inhibitor with anti‐biofilm and anti‐supercoiling properties could counteract bacterial stress responses to antibiotics, in addition to exerting its own antibacterial activity. However, the potential anti‐biofilm and anti‐supercoiling effects of the pioneering compounds, BDFs and SDs, have yet to be described.

Inspired by the cross‐species nature of the phage HU inhibitor Gp46, we conducted a structure‐based drug screening using the two HU major binding sites of phage protein, which led to the identification of a group of HU inhibitors that can be classified as cinnamic‐hydroxamic‐acid derivatives (CHADs). This group of small molecules includes commercialized anti‐cancer drugs Belinostat (trade name Beleodaq, also known as PXD101)^[^ ^34^ ^]^ and Panobinostat (trade name Fardak),^[^ ^35^ ^]^ investigational drugs Dacinostat (LAQ824)^[^ ^36^ ^]^ and Parcinostat (SB939)^[^ ^37^ ^]^ and other CHADs that share the same functional group. We demonstrate that CHADs target the highly conserved regions of HU proteins and displace DNA from HUs in vitro. They inhibit bacterial growth by causing a phenotype similar to that of Gp46 overexpression, i.e., an elongated cell shape in the bacilli, inheriting the property of their phage protein template. In *Staphylococcus aureus* (*S. aureus*), it causes nucleoid loss in the cocci, different from the phenotype in *Bacillus subtilis* (*B. subtilis*) and that of FtsZ inhibitors.^[^ ^38^ ^]^ They are effective against the laboratory strains and clinically separated multidrug‐resistant bacteria strains, including 50 clinically isolated methicillin‐resistant *S. aureus* (MRSA) strains, *M. tuberculosis*, including H37Rv, and an extensively drug‐resistant tuberculosis (XDR‐TB) strain, and *Acinetobacter baumannii* (*A. baumannii*). Due to the roles of HU in biofilm formation and supercoiling, these HU inhibitors also benefit from their anti‐biofilm and supercoiling relaxation properties. With their cytotoxicity and antibacterial activity verified in the in vivo animal models, CHADs have the potential to be used as stand‐alone antibiotics or as adjuvants with other antibiotics.

## 2 Results

### 2.1 Phage‐Inspired and Structure‐Based Screening for HU Inhibitors

Targeting either the “pit” or the α‐helical region of the HUs, SDs, and BDFs have been developed to intervene in the DNA binding properties of their target HUs (Figure S1A–C, Supporting Information). However, SPO1 phage protein Gp46 binds to *B. subtilis* HU (HBsu) via two major interfaces ‐ the edge of the “pit”, i.e., the outermost β sheets (site 1), and the hydrophobic patches (site 2) on two arms (Figure S1D, Supporting Information), different from SDs and BDFs. To identify the most conserved residues within the two target sites for broad‐range inhibitor development, we performed multiple sequence alignment using Clustal Omega^[^ ^39^, ^40^ ^]^ with HU proteins from the ESKAPE pathogens (*Enterococcus faecium, Staphylococcus aureus, Klebsiella pneumoniae, Acinetobacter baumannii, Pseudomonas aeruginosa*, and *Enterobacter* spp.), *B. subtilis*, *E. coli*, *M. tuberculosis*, and the eukaryotic parasite *Plasmodium falciparum* (Figure S1E, Supporting Information). In site 1, most residues (shown for the HBsu) are highly conserved, and in site 2, the hydrophobic and charged residues are highly conserved, which include R58, R61, and I71. Using these two interfaces for targeting site selection, we first virtually screened compound libraries originating from DrugBank^[^ ^41^ ^]^ and ZINC20^[^ ^42^ ^]^ using AutoDock Vina^[^ ^43^ ^]^ (Tables S1–S4, Supporting Information). In addition, the compound protein interaction (CPI)^[^ ^44^ ^]^ score was also used along with the virtual docking to rank the DrugBank molecules (Tables S1 and S2, Supporting Information). The docking results suggest that most highly ranked molecules occupy the key sites (Figure S1F–H, Supporting Information). In site 1, most molecules interact with G46 and K80, which are highly conserved across species. In site 2, all molecules dock with the hydrophobic patch anchored on the highly conserved I71.

### 2.2 Belinostat Is a HU Inhibitor and Causes Nucleoid Deformation in *B. subtilis* and *S. aureus*

**Figure 1:**
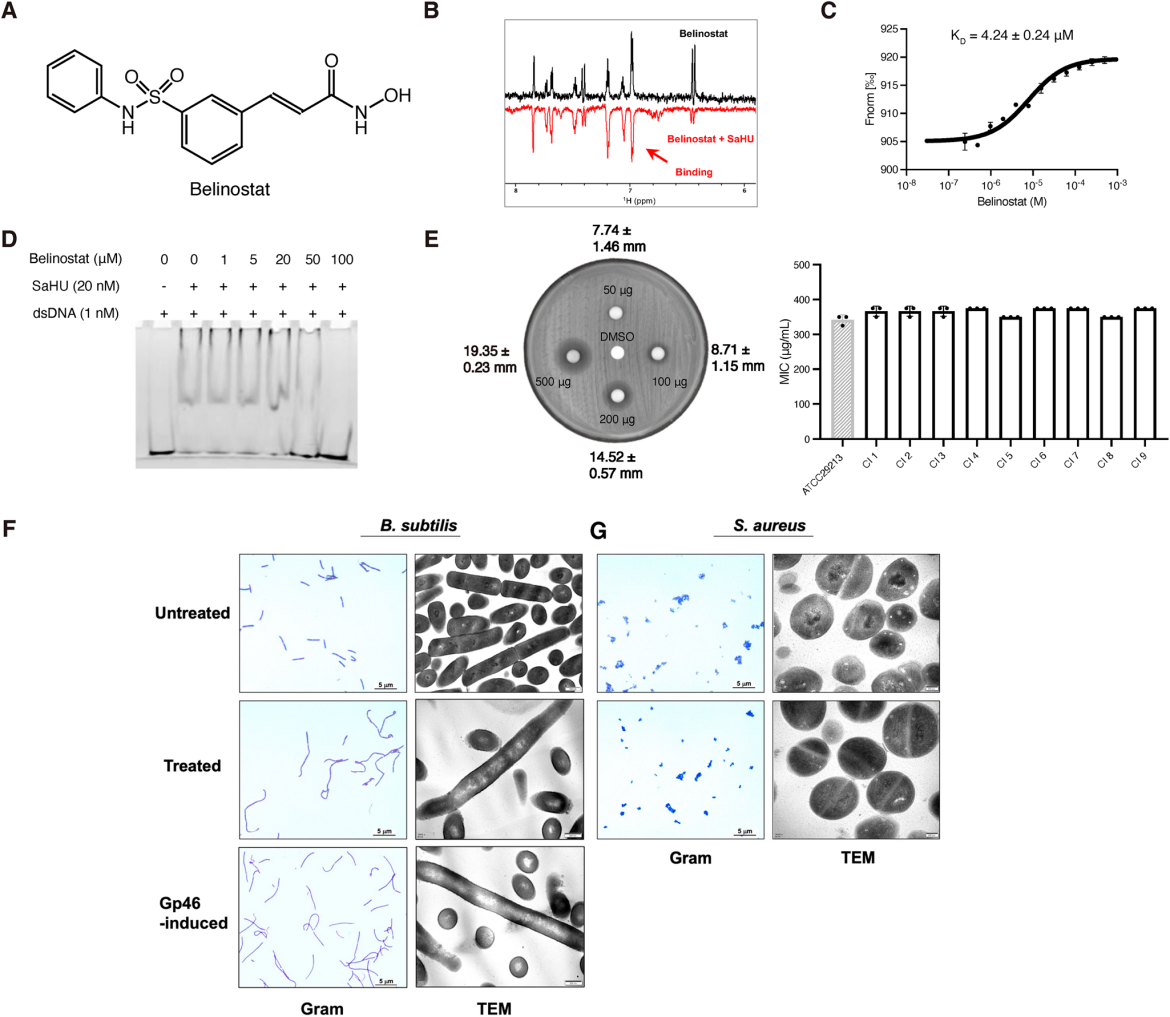
Belinostat is a cross‐species HU inhibitor and a bacterial growth inhibitor. A) The structural formula of Belinostat. B) 1D NMR WaterLOGSY spectra of Belinostat (black) and Belinostat with SaHU (red). C) The MST result of Belinostat‐SaHU interaction. Plots show means, and error bars represent the SEM. D) EMSA showing that Belinostat inhibits SaHU‐dsDNA interaction by replacing the dsDNA out of the SaHU. E) Antimicrobial susceptibility testing (AST) of Belinostat against S. aureus. Left: zone of inhibition assay showing the diameters of the inhibition zones with SD. Right: minimum inhibitory concentrations (MICs) determined for S. aureus and nine clinically isolated MRSA strains. Data represent three technical replicates (*n* = 3), and error bars indicate SD. F) The Gram staining and TEM images of *B. subtilis* with or without Belinostat treatment, as well as overexpressing Gp46. Gram staining scale bar: 5 µm. TEM scale bar: 500 nm. G) The Gram staining and TEM images of *S. aureus* with or without Belinostat treatment. The TEM images show the loss of nucleoid structure after the treatment. Gram staining scale bar: 5 µm. TEM scale bar: 200 nm.

To validate the in silico findings, we performed ligand‐observed nuclear magnetic resonance (NMR) experiments using Water Ligand Observed Gradient Spectroscopy (WaterLOGSY). Top candidate compounds from each screen were tested for interactions with the S. aureus HU protein (SaHU). No interaction was detected for the hits from the ZINC20 library. In contrast, Belinostat (**Figure** **1**A)—an FDA‐approved drug for the treatment of hematological malignancies and solid tumors—exhibited a clear interaction with SaHU in the WaterLOGSY experiment (Figure 1B). Further NMR experiments suggest that Belinostat interacts with the HU proteins of another 5 different bacteria, including *A. baumannii, S. pneumoniae*, *B. subtilis*, *M. tuberculosis* and *S. pyogenes* (Figure S2A, Supporting Information). The binding affinity (K_D_) for Belinostat and SaHU was determined at 4.24 ± 0.24 µm (using 1:1 fitting model) using MicroScale Thermophoresis (MST) (Figure 1C). The high salt buffer (500 mM NaCl) used in MST to reduce the capillary retention could potentially affect the electrostatic interactions between Belinostat and SaHU, which may result in reduced affinity. The K_D_ is however comparable to the HU inhibitor SD1 but weaker than SD4, whose binding affinities to HU (*M. tuberculosis*) are 1.3 ± 0.2 µm and 0.098 ± 0.013 µm, respectively.^[^ ^14^ ^]^ To check if Belinostat is a SaHU inhibitor that disrupts the SaHU‐DNA interaction in vitro, we performed an electromobility shift assay (EMSA). The result shows that the anti‐cancer drug was able to displace double‐stranded DNA (dsDNA) from SaHU (Figure 1D). Using antibiotic susceptibility testing (AST), we assessed and confirmed the antimicrobial activity of Belinostat against *S. aureus* (ATCC 29213 if not stated otherwise). Following the protocols and parameters set by CLSI standards,^[^ ^45^ ^]^ we determined the average minimal inhibitory concentrations (MICs) for the laboratory strain and nine other clinically isolated MRSA (Figure 1E) to be 364 µg mL^−1^ (1.14 mm), which is comparable to both SD1 and SD4, at 400 and 800 µm, respectively. Furthermore, we showed that Belinostat also inhibits the growth of the Gram‐positive model bacterium *B. subtilis* and the MICs for the two strains (ATCC 6051 and 168) were ≈360 µg mL^−1^ (≈1.13 mm) (Figure S2B, Supporting Information), but has little inhibitory effect against *Pseudomonas aeruginosa* (*P. aeruginosa*, PAO1 if not stated otherwise) (Figure S2C, Supporting Information). This observation is consistent with reports on other HU inhibitors, as the functions of HU can be partially compensated in bacteria such as *E. coli* and *P. aeruginosa* by additional nucleoid‐associated proteins (NAPs) with overlapping functions encoded in their genomes.^[^ ^14^, ^46^ ^]^

A distinctive phenotype caused by HU inhibition, as demonstrated by overexpression of the phage protein Gp46 in its native host B. subtilis, is cell elongation accompanied by nucleoid deformation.^[^ ^17^ ^]^ To check if the effects of Belinostat are similar, we examined the phenotypes of *B. subtilis* (strain 168) and *S. aureus* that were treated with 400 µg mL^−1^ (1.26 mm) of Belinostat, assessing the impact of CHAD on the morphology of bacilli and cocci, respectively. Belinostat‐treated *B. subtilis* cells showed diffused nucleoid structures and elongated body shapes in both Gram staining and transmission electron microscopy (TEM) observations, resembling the phenotype caused by overexpression of Gp46 (Figure 1F). For Belinostat‐treated *S. aureus*, there was no noticeable difference in Gram staining, however, TEM revealed that Belinostat‐treated *S. aureus* showed a deformed nucleoid structure (Figure 1G). These observations suggest that the inhibition of bacterial HU is a major contributor to the antibacterial activity of Belinostat.

### 2.3 Identification of CHADs as a Class of Antibacterial Compounds

**Figure 2:**
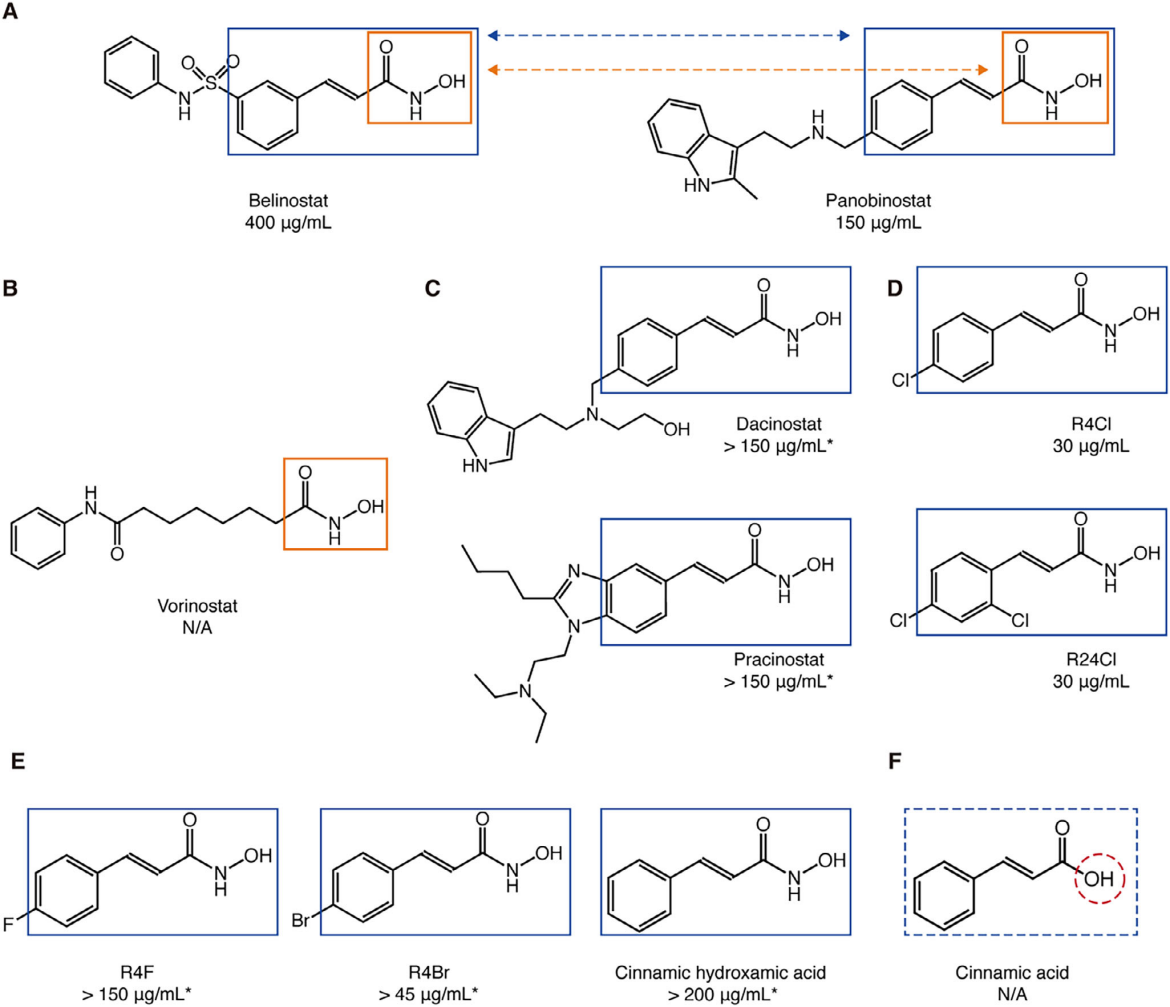
CHADs are cinnamic‐hydroxamic‐acid derivatives. A) The commercially available anti‐cancer drug: Belinostat and Panobinostat ‐ with the shared HA and CHAD groups indicated by orange and blue square, respectively. B) The HDAC inhibitor Vorinostat contains only HA group. C) Two HDAC inhibitors (phase 1 clinical trial passed) ‐ Dacinostat and Pracinostat that contain HA and CHAD groups. D) R4Cl and R24Cl representing adding R groups to CHA. E) The structural formulas of R4F, R4Br, and CHA. F) The structural formula of cinnamic acid. * Maximum solubility reached.

We next questioned whether the identification of Belinostat as an anti‐bacterial agent was an isolated case or could the functional group responsible for its anti‐microbial activity serve as the scaffold for optimization. Reports in the literature indicate that histone deacetylase inhibitors (HDACi) may be effective in treating bacterial infections by modulating human defense systems.^[^ ^47^, ^48^, ^49^ ^]^ This suggests that the hydroxamic acid (HA) or cinnamic hydroxamic acid (CHA) groups, shared by these non‐selective HDACis, could contribute to their antimicrobial activity. We used the Pangu Molecule Optimizer (PGMO) in an attempt to expand our lead compound library.^[^ ^44^ ^]^ By choosing the different functional groups as scaffolds, we generated a collection of derivatives of Belinostat (Table S5A,B, Supporting Information). Although the results of PGMO did not contain any commercialized drugs, some of the top optimized small molecules possess a cinnamic hydroxamic acid (CHA) group, which resembles another FDA‐approved drug Panobinostat, which is also a HDACi (Figure 2A; Figure S3A, Supporting Information). Importantly, both Panobinostat and Belinostat are also CHADs. Using S. aureus as a representative clinically significant pathogen, we showed that Panobinostat also exhibits antimicrobial activity, with a MIC of 150 µg mL^−1^ (430 µm) (Figure S3B, Supporting Information). To determine if the CHA group is essential for the antimicrobial activity, we selected another HDACi, Vorinostat (trade name Zolinza and Figure 2B),^[^ ^50^ ^]^ which only has the HA group, and demonstrated that it did not interact with SaHU nor inhibit the growth of *S. aureus* (Figure S3C–E, Supporting Information). Furthermore, two further HDAC inhibitors, Dacinostat^[^ ^36^ ^]^ and Parcinostat,^[^ ^37^ ^]^ also showed antibacterial activity against *S. aureus* (Figure 2C; Figure S3C, Supporting Information). In summary, the CHA group is likely to be the key functional group responsible for the antimicrobial activity of HDAC inhibitors.

To confirm whether CHA is responsible for the anti‐bacterial properties, we defined the CHA group as the scaffold base and searched ZINC20 for its derivatives. A collection of small molecules that contains the CHA group was identified (Figure 2D,E; Figure S4A,B, Supporting Information). Two molecules that contain one or two chlorine atoms as the R group of CHA, termed R4Cl and R24Cl, respectively, showed antibacterial properties in the AST (Figure 2D; Figure S4A, Supporting Information). We then determined the MICs of R4Cl and R24Cl for *S. aureus*, both to be ≈30 µg mL^−1^ (152 µm of R4Cl and 130 µm of R24Cl) (Figure S4B, Supporting Information). Compared with Belinostat or Panobinostat, R4Cl and R24Cl have significantly lower MICs, suggesting the chlorine substitution at the R4 position of the CHA scaffold has an important role in the antimicrobial properties. To examine the role of the R4 chlorine, we synthesized two molecules that have halogen substitutions, namely R4F and R4Br (Figure 2E). Interestingly, R4F partially inhibited the growth of *S. aureus* at 150 µg mL^−1^ (828 µm), and R4Br has a higher MIC than R4Cl, at 60 µg mL^−1^ (248 µm) (Figure S4A,C, Supporting Information). MST experiments to determine the affinities between R4 substitutes and SaHU had large errors, suggesting that the high salt may interfere with the interaction. Furthermore, CHA, without any R4 substitution, also showed antibacterial ability (Figure 2E; Figure S4A,D, Supporting Information). However, it only partially inhibited the growth of *S. aureus* at 200 µg/mL (1.23 mM). Interestingly, its binding affinity to SaHU (5.77 ± 0.93 µm) determined by MST (fitting model 1:1) is similar to that of Belinostat (Figure S4E, Supporting Information). Taken together, these substitutions suggest that the R4 group is important for the antibacterial properties of the CHADs. And a list of proposed CHADs that may have potential antibacterial activities is shown in Figure S5A,B (Supporting Information). Meanwhile, to examine whether the HA group of the CHA scaffold has a role in the antibacterial activity, we explored cinnamic acid, representing the removal of the amide from the HA group. As cinnamic acid lost the anti‐bacterial activity (Figure S6A,B, Supporting Information), CHA is therefore the functional group for antibacterial properties of these molecules.

### 2.4 CHADs Target Site 1 of SaHU

**Figure 3:**
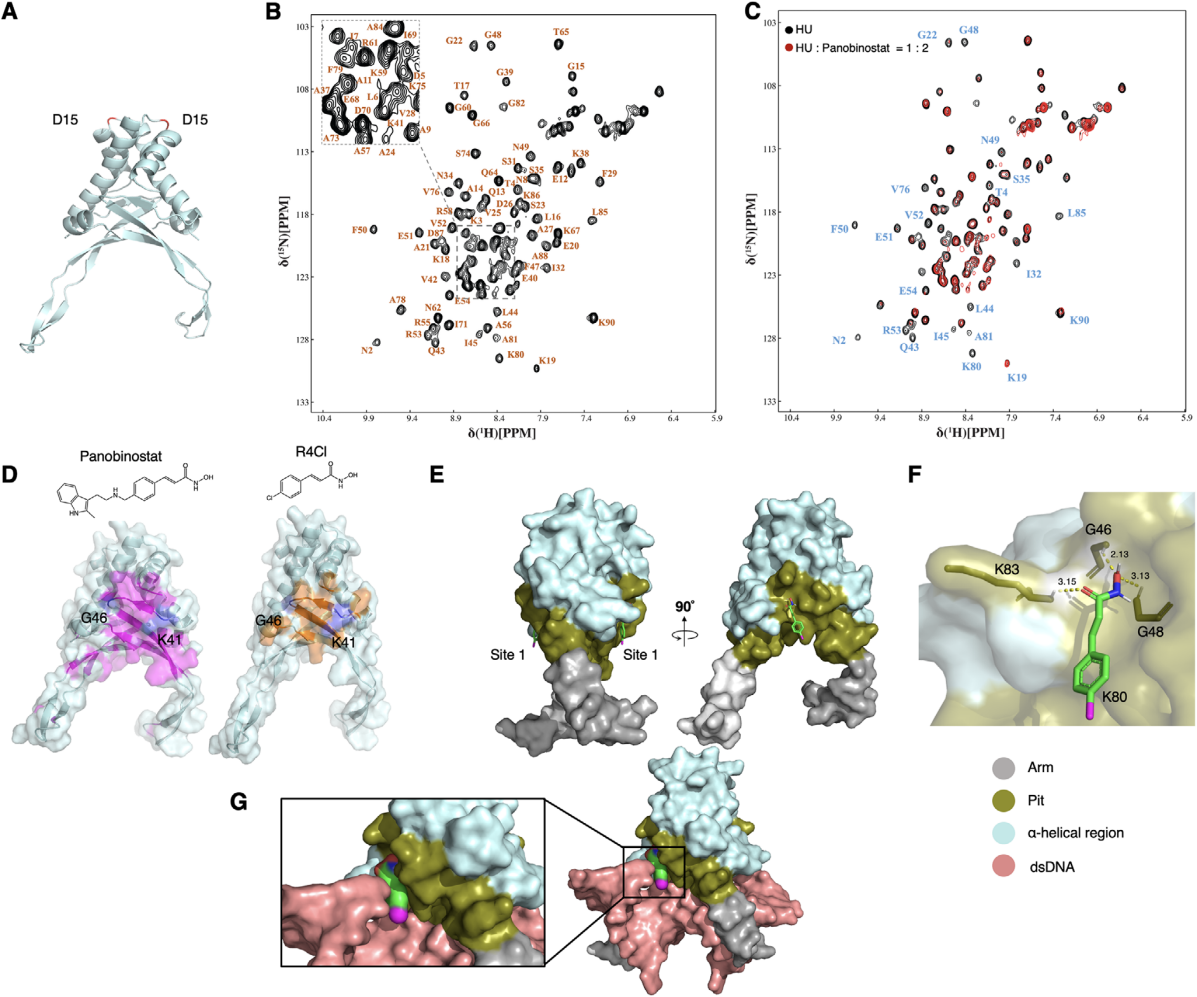
CHADs target site 1 of SaHU. A) The crystal structure of SaHU with D15 is coloured in red. B) The NMR backbone assignment of SaHU^D15G^ mutant. C) Overlay of ^1^H‐^15^N HSQC spectra of SaHU^D15G^ (black), and SaHU^D15G^ with 2 molar equivalents of Panobinostat added (red). D) After Panobinostat treatment (left), the exposed residues of SaHU^D15G^ that experienced a significant peak broadening effect, as demonstrated in C) and Figure S7A (Supporting Information) are highlighted in pink on the structure. After R4Cl treatment (right), the exposed residues of SaHU^D15G^ that experienced significant peak broadening effect as demonstrated in Figure S7B,C (Supporting Information) are highlighted in orange. G46 (not assigned) and K41 (untraceable) are highlighted in blue. E) Proposed SaHU‐CHAD complex model (2:2). F) H‐bond network between R4Cl and G46, G48, and K83 in the proposed model. G) Proposed mechanism of competition between CHADs and DNA for HU binding.

To elucidate the antibacterial mechanism of CHADs at the molecular level, we sought to obtain a structure of the HU‐CHAD complex. Using S. aureus HU as a representative, attempts to crystallize the SaHU‐CHAD complex were unsuccessful. Instead, we obtained a 2.14 Å crystal structure of apo SaHU (**Figure** **3**A; PDB ID: 8HD5) and proceeded to generate an NMR‐derived model for the complex. To facilitate the structural study for the complex, we constructed a mutant SaHU^D15G^ to stabilize the protein in solution following the previous studies provided by a HU homologue.^[^ ^51^ ^]^ Using standard NMR triple‐resonance approaches, we obtained a full backbone assignment with only G46 and K19 unannotated (Figure 3B). Using Panobinostat to represent the commercialized drugs and R4Cl for the new molecules, we next performed NMR titration experiments against SaHU^D15G^. Many peaks in ^1^H‐^15^N HSQC spectrum of ^15^N‐labeled SaHU^D15G^ exhibited significant peak broadening when 2 molar equivalents of Panobinostat were added (Figure 3C). We plotted the changes in peak intensity against residue number (Figure S7A, Supporting Information) and projected these changes on the structure (Figure 3D, left). The line‐width perturbations were mostly observed in the site 1 and its surrounding residues, indicating the drug binds to site 1 as determined from the virtual screening. A similar set of residues (N2, K18, V42, Q43, and E51‐E54) also experienced peak broadening, albeit less significantly when R4Cl was added, indicating the underlying mechanism of interactions are likely to be similar, i.e., targeting site 1 of SaHU^D15G^ (Figure 3D, right; Figure S7B,C, Supporting Information). In the NMR titration experiment of CHA, some of the peaks (N2 and E51) that experienced the peak broadening in the previous titrations showed chemical shift perturbations instead (Figure S7D, Supporting Information), indicating a weaker interaction.

Using the active interfacial residues of SaHU^D15G^ identified from NMR titration, together with the CHA group from R4Cl, we modeled the R4Cl–SaHU^D15G^ complex using the High Ambiguity Driven protein–protein Docking (HADDOCK) server (Figure 3E).^[^ ^52^ ^]^ The CHAD positions at the site 1 of SaHU^D15G^ with the HA group pointing toward α‐helical region of SaHU^D15G^ forming multiple H‐bonds with surrounding residues. Meanwhile, the ring of R4Cl is located near K80, and the sidechain on the ring is extending toward the pit region of SaHU^D15G^, which would otherwise be occupied by DNA. Amino acid donors G46, G48, and K48 form multiple hydrogen bonds with the HA group of R4Cl, while the side chain of K80 interacts with the ring via hydrophobic and electrostatic interactions in the model (Figure 3F). By occupying site 1 of HU, R4Cl or other CHADs would block the HU‐DNA interaction. This is highlighted in the structure of the SaHU‐DNA complex (PDB code 4QJU, Figure 3G). The model indicates that any additional substituents on the ring that extend toward the pit region of HU would interfere with the interaction between DNA and SaHU. The extension can be a bulky group, as seen in Belinostat or electron withdrawing group as seen in R4Cl.

To evaluate the complex, we also employed molecular dynamics (MD) for the free energy calculation for the SaHU^D15G^‐R4Cl complex. The result suggests that R4Cl attached to the binding site throughout the simulation with an absolute binding free energy of 13.36 kcal mol^−1^ (Video S1, Supporting Information). The estimated energy contribution per residue to the binding suggests that residues L44, I45, G46, K80, G82, and K83 contribute most to the free energy (Figure S8A,B, Supporting Information). Furthermore, we calculated the relative binding free energy (RBFE, ΔΔG) for all the MIC‐determined molecules shown in Figure 2 based on the SaHU^D15G^‐R4Cl model with Belinostat as the reference compound. A plot of the experimentally determined MICs of these molecules against the calculated ΔΔG shows a positive correlation, with only Panobinostat as the only outlier (Figure S8C, Supporting Information). The agreement between the MD‐based calculations and experimentally determined MICs suggests the NMR‐derived mode of the complex would be useful for further optimization of the CHADs.

### 2.5 The Antibacterial Property of CHADs

**Figure 4:**
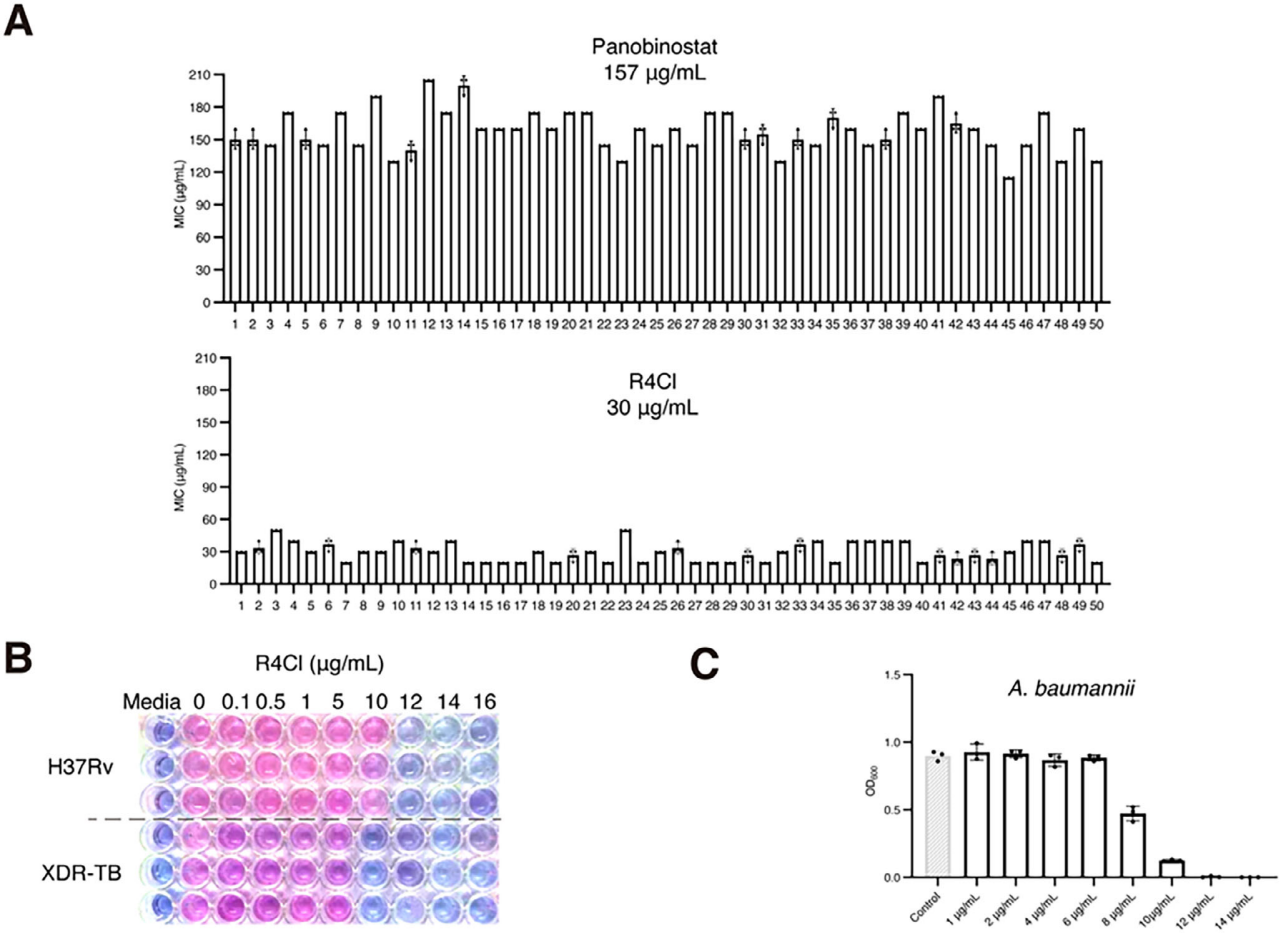
CHADs are effective against Gram‐positive bacteria and some Gram‐negative bacteria. A) The MICs of Panobinostat (upper) and R4Cl (lower) for 50 clinically isolated MRSA. Plots show three technical replicates (*n* = 3), and error bars represent the SD. B) The MICs of R4Cl for *M. tuberculosis* and an XDR‐TB strain using Resazurin microtitre plate assay. C) The MIC determination of R4Cl for *A. baumannii*. Plots show three technical replicates (*n* = 3), and error bars represent the SEM.

To verify if CHADs are effective against drug‐resistant bacterial strains, we chose Panobinostat from the commercialized drug group and R4Cl from the new molecule group to represent the drug repurposing route and new drug development. We focused on the clinically significant bacteria in which HU is indispensable, including *S. aureus*, *M. tuberculosis*, and A. *baumannii*. First, Panobinostat and R4Cl were tested against 50 clinically isolated MRSA strains and two strains of M. tuberculosis. The 50 MRSA strains were collected from local hospitals, and their multidrug‐resistant profiles are shown in Figure S9A (Supporting Information). Whole‐genome sequencing and phylogenetic analysis revealed that none of the strains were identical (Figure S9B, Supporting Information). The average MIC of Panobinostat against the 50 MRSA stains is 157 µg mL^−1^ (449 µm) (**Figure** 4A, upper). And R4Cl exhibited an average MIC of 30 µg mL^−1^ (152 µm) (Figure 4A, lower), which is significantly lower than that of Panobinostat. The resazurin microtiter plate assay suggested R4Cl inhibited the growth of both *M. tuberculosis* (H37Rv if not stated otherwise) and an XDR‐MTB strain (the multidrug‐resistant profile can be found in Table S6, Supporting Information), and the MICs for XDR‐TB and *M. tuberculosis* are 10 and 12 µg mL^−1^ (51 and 61 µm) (Figure 4B). Similarly, the MIC determined for A. *baumannii* (BBA‐1605 if not stated otherwise) is also around 12 µg mL^−1^ (61 µm) (Figure 4C). Furthermore, the frequency of resistance (FoR) to R4Cl was found to be 1 × 10^−10^ for the *S. aureus*. Given that HU plays active roles in bacterial supercoiling and biofilm formation, both of which contribute to bacterial resistance, its inhibitors would potentially have the advantage of a low FoR.

### 2.6 Toxicity Tests

To demonstrate the potential of CHADs in antibacterial applications, we selected two commercial anti‐cancer drugs, Belinostat and Panobinostat, along with one of the best‐performing new molecules, R4Cl, for safety evaluations in mice. For skin applications, the three drugs were applied to the skin of mice for seven consecutive days at a concentration of 1000 mg/kg. No morphological abnormalities were observed in the heart, liver, kidney, lung, or spleen tissues in any of the drug‐treated groups compared to the control (Figure S10, Supporting Information).

**Figure 5:**
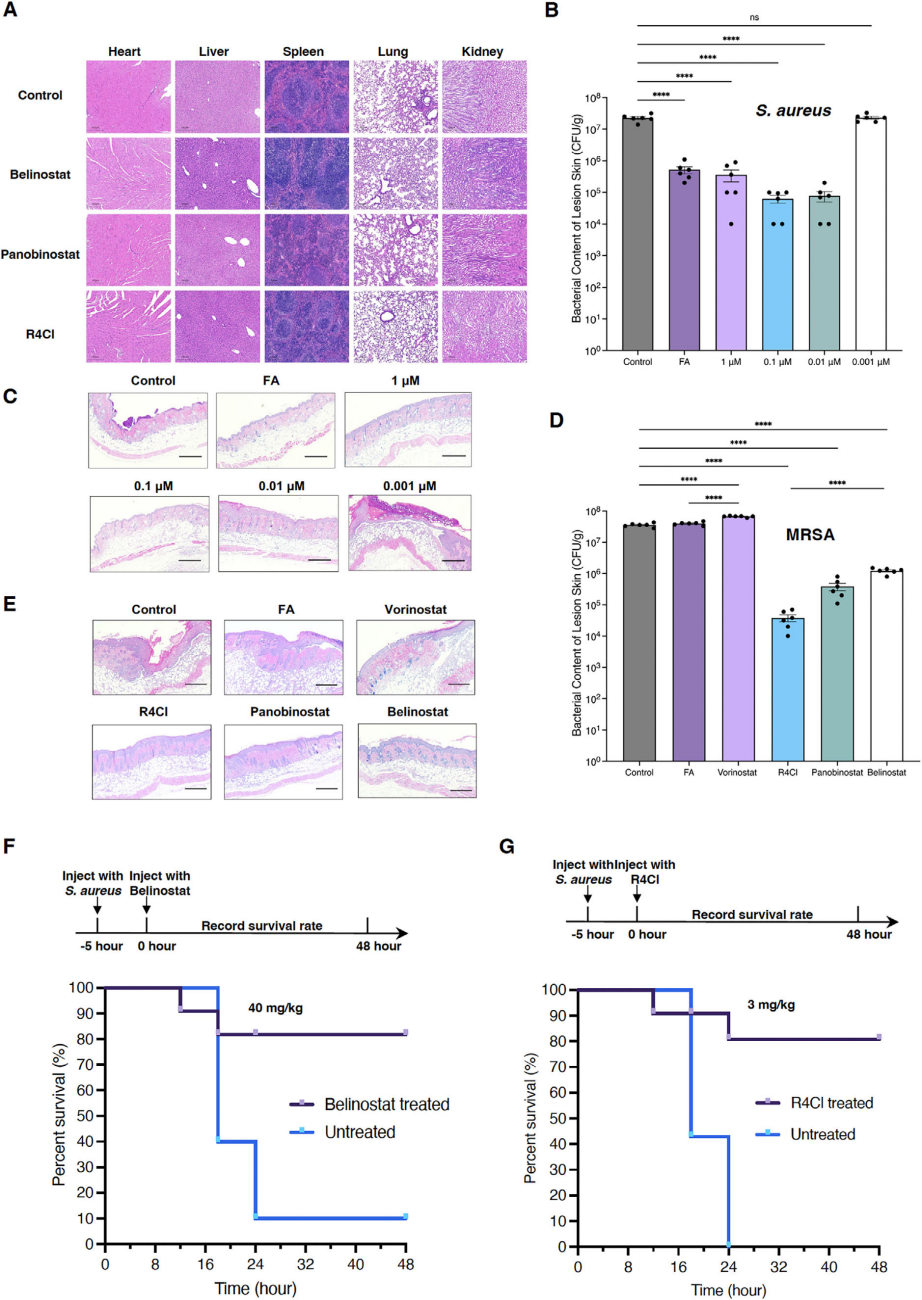
The effectiveness of CHADs in mice models. A) Histopathological assessment of heart, liver, kidney, lung, and spleen tissues in mice after injection of Belinostat (44 mg kg^−1^), Panobinostat (28 mg kg^−1^), R4Cl (44 mg/kg) or negative control buffer 7 days. Scale bar: 100 µm. B) Bacterial counts (CFU/g) in *S. aureus* infected mice skins after treatment with negative control buffer, FA (2%) and Belinostat (0.001, 0.01, 0.1 and 1 µm), respectively. Statistical significance determined by one‐way ANOVA. Plots show the individual values for each mouse (*n* = 6), and error bars represent the SEM. ^****^ *p* < 0.0001, and ns denotes not significant compared to control. C) Histopathological assessment of *S. aureus* infected mice skins after treatment with negative control buffer, FA (2%) and Belinostat (0.001, 0.01, 0.1, and 1 µm), respectively. Scale bar: 200 nm. D) Bacterial counts (CFU/g) in FA‐resistant MRSA infected mice skins after treatment with negative control buffer, FA (2%), Vorinostat, R4Cl, Panobinostat, and Belinostat, respectively. Statistical significance determined by one‐way ANOVA. Plots show the individual values for each mouse (*n* = 6), and error bars represent the SEM. ^****^ *p* < 0.0001. E) Histopathological assessment of FA‐resistant MRSA infected mice skins after treatment with negative control buffer, FA (2%), Vorinostat, R4Cl, Panobinostat, and Belinostat, respectively. Scale bar: 200 nm. F) Kaplan‐Meier survival curves (*n* = 10) of the solvent used in this study (untreated) and Belinostat (40 mg kg^−1^) injected *S. aureus* sepsis mice that monitored for 48 h. (G) Kaplan‐Meier survival curves (*n* = 10) of the solvent used in this study (untreated) and R4Cl (3 mg kg^−1^) injected *S. aureus* sepsis mice that monitored for 48 h.

For intravenous injection, the median lethal dose (LD_50_) was determined using the up‐and‐down procedure to assess acute toxicity.^[^ ^53^ ^]^ The LD_50_ values for Belinostat and R4Cl were both determined to be 242.7 mg kg^−1^. However, Panobinostat, which is only approved for oral administration, has a lower LD50 of 78.64 mg kg^−1^ (Figure S11A–C, Supporting Information). These LD_50_ values suggest that R4Cl has a similar toxicity level to Belinostat. Additionally, the three drugs were subjected to toxicity tests in mice for seven consecutive days via daily intravenous injections. Belinostat and R4Cl were administered at a daily dose of 44 mg kg^−1^, while Panobinostat was given at 28 mg kg^−1^ due to its higher toxicity. After 7 days, the mice were sacrificed. Blood, heart, liver, kidney, lung, and spleen tissues were harvested, and tissue sections were stained with hematoxylin‐eosin (H&E) for histopathological assessment. No morphological abnormalities were observed, except for the lung tissues treated with Belinostat and Panobinostat, which showed moderate neutrophil infiltration and alveolar wall thickening, indicative of lung injury (**Figures** **5**A and S12A, Supporting Information). Furthermore, significant reductions in white blood cells (WBCs), neutrophils (Neu), lymphocytes (Lym) and platelets (PLT), were found in the Panobinostat‐treated group compared to the control, which is consistent with the reported side effects of Panobinostat.^[^ ^54^ ^]^ There were no observable differences in the number of red blood cells (RBCs) or haemoglobin (HGB) levels among the three drug groups (Figure S12B, Supporting Information). These results support the fact that Belinostat is safe for intravenous injection and Panobinostat is unsuitable for intravenous injection, as well as show that R4Cl has similar pharmacological efficacy to Belinostat. However, further studies are needed to fully assess the pharmacokinetic characteristics of R4Cl.

### 2.7 CHADs Are Effective in Treating S. aureus Infections in Both Skin Infection and Septic Mice Models

To determine whether the in vitro effect could be translated in vivo, we next evaluated the CHADs in direct comparison with fusidic acid (FA) using a skin infection model. Superficial wounds on the back of mice were infected with *S. aureus* and treated with FA or Belinostat. Compared to the control mice, the bacterial content per gram of lesion skin (CFU/g) were significantly reduced after treatment with FA (2%) or concentrations of Belinostat between 3 ng mL^−1^ (0.01 µm) and 300 ng mL^−1^ (1 µm), which is notably smaller than the measured MIC (Figure 5B). Consistent with these observations, the control group showed severe lesions in epidermis layer and inflammatory cell infiltration in the subcutaneous tissue, but FA or Belinostat‐treated groups led to restored epidermis and reduced inflammation (Figure 5C). We extended the comparison to include three more CHADs (0.1 µm), including Panobinostat, R4Cl, and a HDACi ‐ Vorinostat (also 0.1 µm), in the FA‐resistant MRSA infection model. All three CHADs were effective in clearing the infection in the skin lesion, and marked regenerative changes were observed (Figure 5D,E). On the other hand, FA or Vorinostat did not inhibit bacterial propagation with intense inflammatory cell infiltration in the subcutaneous tissues. The comparative study suggests that CHADs are effective in treating superficial infections but HDACi ‐ Vorinostat that does not contain the CHA functional group, was unable to suppress the infection yet even promoted the infection. And the R4Cl which has the lowest MIC among the CHADs performed best, whilst the Belinostat which has the highest MIC, performed worst in the bacteria count (Figure 5D). This emphasizes the fact that the antibacterial activities of the CHADs, can be translated into the animal model.

Furthermore, to evaluate the systemic antibacterial effects of CHADs, we tested Belinostat in mouse models of S. aureus and P. aeruginosa sepsis, using the latter for comparison since CHADs exhibit minimal in vitro activity against P. aeruginosa. Belinostat has been approved for intravenous injection to treat peripheral T‐cell lymphoma, and has been proven to be safe for mice at 40 mg kg^−1^ with a C_max_ of around 50 µg mL^−1^ in pre‐established in vivo surrogate models, such as patient‐derived xenografts (PDX).^[^ ^55^ ^]^ Within 48 h, the survival rates of *S. aureus‐* and *P. aeruginosa*‐septic mice injected with the solvent used in this study were 10%. However, Belinostat was able to rescue *S. aureus‐*treated mice with a survival rate of 90% at 16 h and maintain it at 80% for 48 h (Figure 5F). In contrast, it had no effect on the survival rate at 32 h of *P. aeruginosa*‐treated mice with groups at 10%, albeit the drug did extend the survival time by around 8 h (Figure S13A, Supporting Information). As Belinostat displays only a small reduction in the growth of *P. aeruginosa* (Figure S2C, Supporting Information), our data further support the notion that AST results can be translated into the in vivo models. Importantly, Belinostat could be considered as an emergency treatment for septic patients with *S. aureus* infection. Furthermore, the survival assay was repeated using R4Cl at 3 mg kg^−1^, based on its lower MIC (30 µg mL^−1^ or 152 µm) than Belinostat (400 µg mL^−1^ or 1.26 mm). The reduced dose of R4Cl was able to rescue *S. aureus‐*treated mice with the survival rate 80% after 48 h (Figure 5G). In conclusion, our mice models agree with a previous study showing some HDACs impair host defenses to bacterial infection,^[^ ^48^ ^]^ as Vorinostat‐treated mice suffered more serious infections than the control in the skin infection model. And R4Cl, which inherits low toxicity from its parental template Belinostat, has improved antibacterial activity in vivo. Notably, there is a discrepancy between the MIC determined in AST and the effective concentrations required in vivo. This could be attributed to the possibility that CHADs become sequestered by extracellular HU during AST, reducing their apparent potency in vitro.

### 2.8 The Anti‐biofilm Property of CHAD

**Figure 6:**
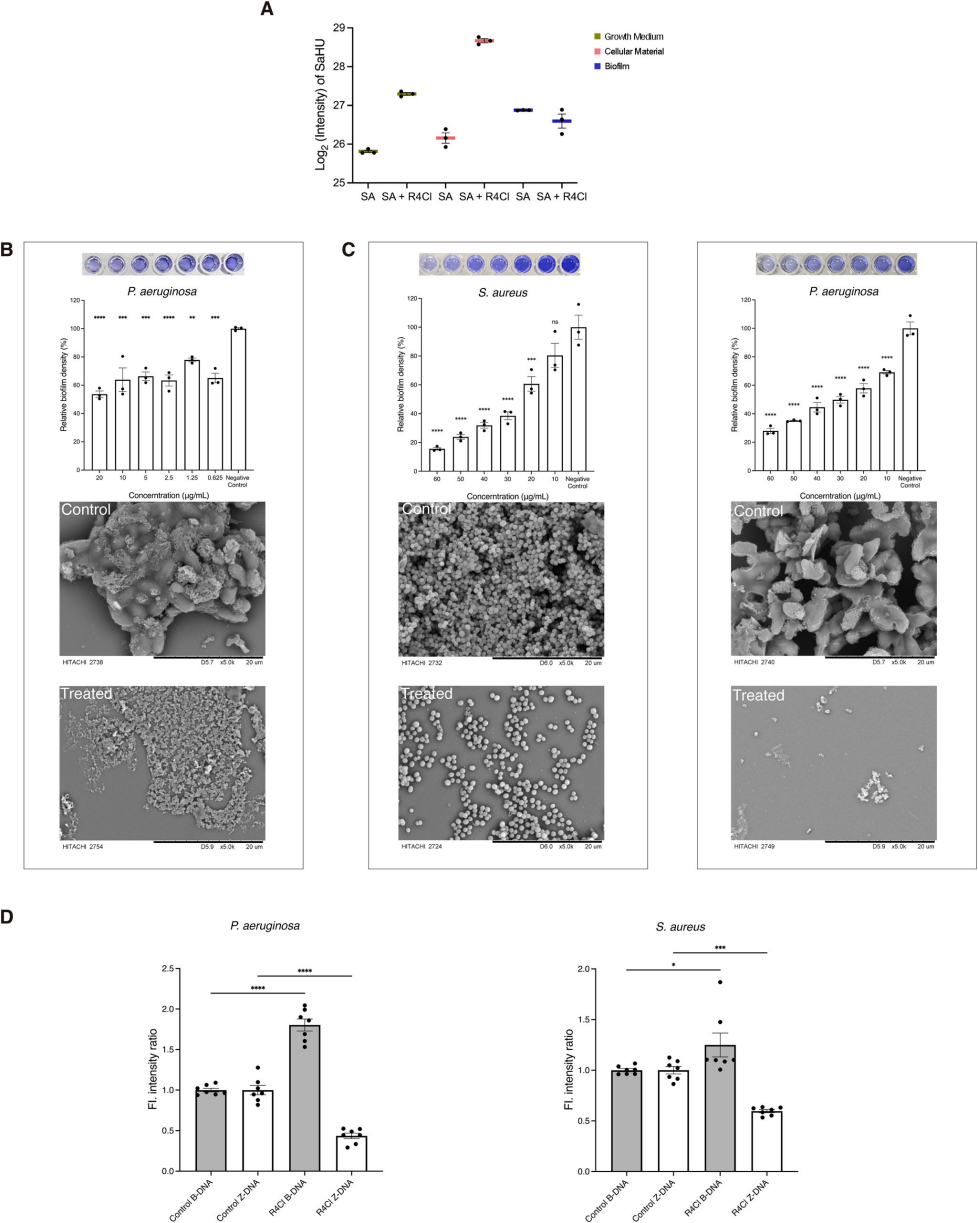
The anti‐biofilm property of R4Cl. A) The Log_2_ (intensity) values of SaHU in different forms determined by MS. Plots show three biological replicates (*n* = 3), and error bars represent the SEM. B) Inhibition of *P. aeruginosa* biofilm. R4Cl was added at the time of bacterial inoculation. Upper panel: the crystal violet assay with the image shown above the chart as a representative. Negative Control (Control): 1% DMSO. Statistical significance determined by one‐way ANOVA. Plots show three technical replicates (*n* = 3), and error bars represent the SEM. ^****^ *p* < 0.0001, ^***^ *p* < 0.001, ^**^ *p* < 0.01, and ns denotes not significant compared to control. Lower panel: the SEM images of the biofilm (Control) and those treated with R4Cl (Treated). Scale bar: 20 µm. C) Eradication of biofilms formed by *S. aureus* and *P. aeruginosa*. R4Cl was added after biofilm formation. Upper panel: the crystal violet assays with the images shown above the charts as representatives. Negative Control (Control): 1% DMSO. Statistical significance determined by one‐way ANOVA Plots show three technical replicates (*n* = 3), and error bars represent the SEM. ^****^ *p* < 0.0001, ^***^ *p* < 0.001, and ns denotes not significant compared to control. Lower panel: the SEM images of the biofilm (Control) and those treated with R4Cl (Treated). Scale bar: 20 µm. D) Changes in the abundance of B‐DNA or Z‐DNA in the presence of 20 µg mL^−1^ (101 µm) R4Cl were quantified as the ratio of their fluorescence intensity (FI) to that of untreated biofilms using ImageJ. FI was normalized to total biomass (FM4‐64 fluorescent signal). Statistical significance determined by unpaired t tests. Plots show seven technical replicates (*n* = 7), and error bars represent the SEM. ^****^ *p* < 0.0001, ^***^ *p* < 0.001, and ^*^ *p* < 0.05.

To assess whether CHAD can be trapped extracellularly, we examined the different forms of HU during in vitro experiments. In the AST, HU exists in three forms: cellular, in the growth medium, and in biofilm. This differs from in vivo models, where an environment to confine extracellular free HU, as seen in culture plates, does not exist, and HU primarily remains intracellular. To determine the distribution of HU in these three forms under in vitro conditions, we treated S. aureus with or without R4Cl and analyzed HU levels in each form using mass spectrometry (MS). The results showed that HU was more abundant in the extracellular forms, particularly in the biofilm, compared to the intracellular form (**Figure** **6**A; Figure S14A, Supporting Information), with the highest HU levels detected in the biofilm. Upon treatment with a subinhibitory concentration of R4Cl (0.5 × MIC, 15 µg mL^−1^), HU remained predominantly extracellular; however, its distribution shifted, with biofilm‐associated HU showing the greatest reduction. Notably, the microbial counts of the treated and untreated cultures were nearly identical, indicating that this shift was not due to differences in bacterial growth. These findings provide an explanation for the discrepancies between in vivo and in vitro observations and suggest that R4Cl may possess anti‐biofilm activity, as evidenced by the decrease in biofilm‐associated HU alongside increases in intracellular and growth medium HU levels.

Although CHADs exhibit minimal in vitro antibacterial activity against P. aeruginosa, they may still inhibit its biofilm formation. To test this, R4Cl was applied at a non‐inhibitory concentration for P. aeruginosa (20 µg mL^−1^ or 101 µm) to avoid interference from its antibacterial activity. When R4Cl was added at the time of bacterial inoculation, the crystal violet staining results suggest that R4Cl inhibits biofilm formation of *P. aeruginosa* at concentrations below 20 µg mL^−1^ (101 µm) (Figure 6B). The scanning electron microscopy (SEM) also shows that the biofilm formation was inhibited. We then used a biofilm eradication assay to verify the potential biofilm‐destructive effect of CHADs by adding R4Cl after biofilm formation. We demonstrated that R4Cl has a strong biofilm‐destructive effect against *S. aureus*, with over 60% destruction at 1 × MIC (30 µg mL^−1^ or 152 µm) and over 80% at 2 × MIC (60 µg mL^−1^ or 304 µm) (Figure 6C). R4Cl exhibited a similar effect against biofilms formed *by P. aeruginosa*, although it is not effective against the bacterium itself. And the SEM results also support the crystal violet staining data, showing the disruption of biofilms performed by *S. aureus* and *P. aeruginosa*.

In biofilms, the architectural Z‐DNA is stabilized by DNABII family proteins, including HU and IHF. Since CHADs disrupt HU‐DNA interactions, they may also destabilize Z‐DNA, contributing to compromised biofilm structural integrity. To monitor potential B‐to‐Z DNA conversion under the influence of R4Cl, we employed B‐DNA‐specific and Z‐DNA‐specific antibodies in biofilms preformed by P. aeruginosa and S. aureus, following previously described methods.^[^ ^26^ ^]^ After normalization to biofilm biomass (FM4‐64 fluorescent signal), we observed a clear shift in both biofilms: the proportion of B‐DNA increased, whereas the Z‐DNA fraction decreased significantly following R4Cl treatment, indicating destabilization of the Z‐DNA architecture (Figure 6D; Figure S14B,C, Supporting Information). Additionally, it appears that the fibre‐like DNA structures were also removed, which is known to be associated with high elasticity for *P. aeruginosa* biofilms.^[^ ^56^ ^]^ These observations are consistent with the role of HU in stabilizing Z‐DNA and demonstrate that R4Cl inhibits HU‐DNA binding.

### 2.9 The Supercoiling Relaxation and the Antibiotic Synergy Effect of R4Cl

**Figure 7:**
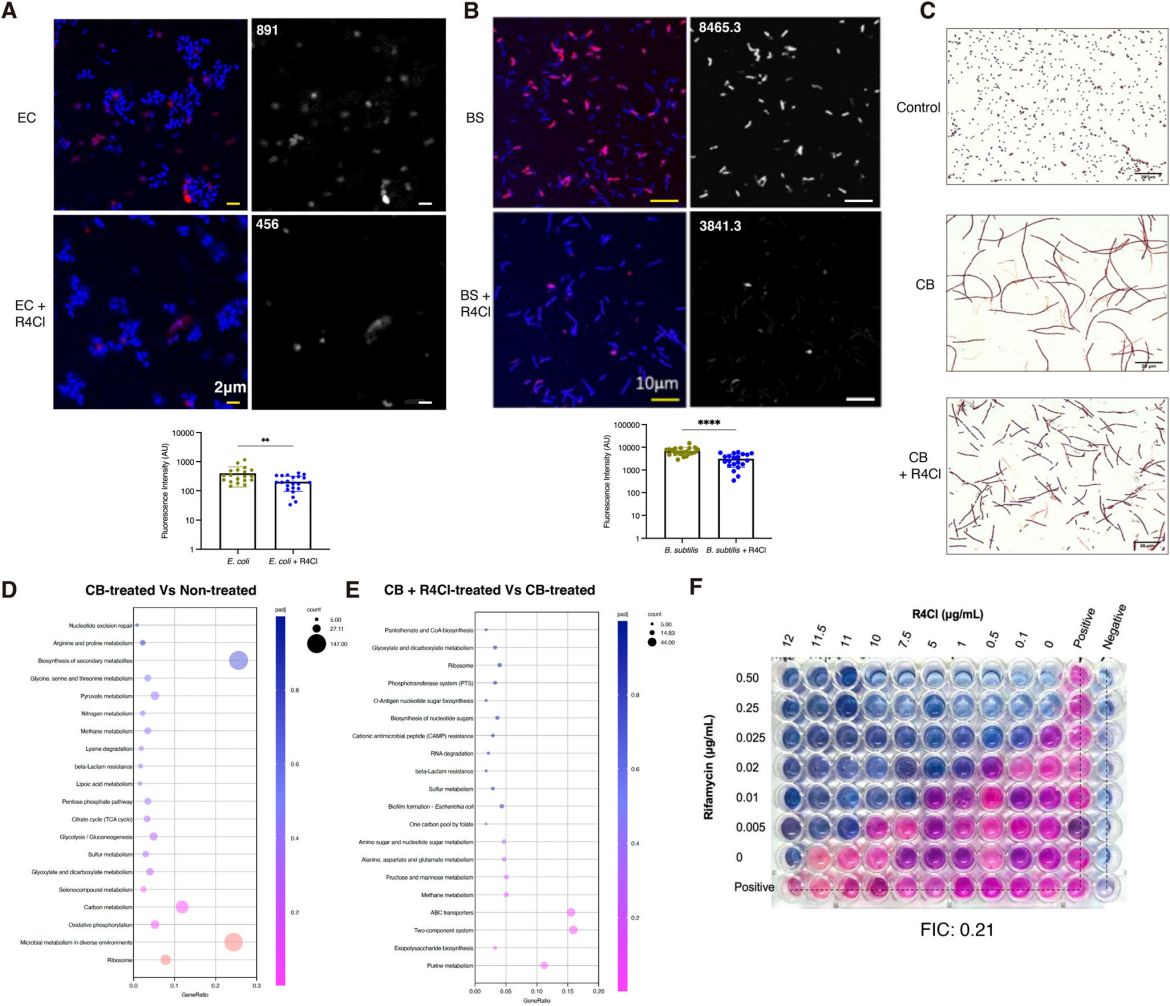
The supercoiling relaxation and the antibiotic synergy effect of R4Cl. A) The representative bTMP staining images of *E. coli* and R4Cl‐treated *E. coli* (top right panels). The number represents the normalized florescence intensity of current frame. Nucleoids were counterstained with DAPI, and merged images were shown (top left panels). Scale bar: 2 µm. The quantification of supercoiling for *E. coli* with or without R4Cl treatment (lower panel). Each point denotes the integrated fluorescence signal from a randomly chosen field. Values are mean ± SEM and are normalized with cell number, *n* = 19. Statistical significance compared to control (*E. coli*) was assessed by unpaired t tests, and ^**^ *P* < 0.01. B) The representative bTMP staining images of *B. subtilis* and R4Cl‐treated *B. subtilis* (top right panels). Both experiments (Figure 7A,B) were performed under 10 µg mL^−1^ of R4Cl, lower than the MIC for *B. subtilis* (30 µg mL^−1^). Therefore, the elongation of *B. subtilis* was not as significant as seen in Figure 1F that using MIC concentration. The number represents the normalized florescence intensity of the current frame. Nucleoids were counterstained with DAPI, and merged images were shown (top left panels). Scale bar: 2 µm. The quantification of supercoiling for *B. subtilis* with or without R4Cl treatment (lower panel). Each point denotes the integrated fluorescence signal from a randomly chosen field. Values are mean ± SEM and are normalized with cell number, *n* = 19. Statistical significance compared to control (*B.subtilis*) was assessed by unpaired t tests, and ^****^ *P* < 0.0001. C) The Gram staining images of *E. coli*, CB‐treated *E. coli*, and *E. coli* treated simultaneously with CB and R4Cl. Scale bar: 20 µm. D) KEGG pathway enrichment analysis of differentially expressed genes between 10 µg mL^−1^ of CB‐treated and untreated *E. coli* cells. *y*‐axis indicates the pathway name; *x*‐axis indicates the gene ratio. The bubble size indicates the number of genes, and the color bar indicates the adjusted p‐value. E) KEGG pathway enrichment analysis of differentially expressed genes between 10 µg mL^−1^ of CB and 10 µg mL^−1^ of R4Cl simultaneously treated and only 10 µg mL^−1^ of CB treated *E. coli* cells. *y*‐axis indicates the pathway name; *x*‐axis indicates the gene ratio. The bubble size indicates the number of genes, and the color bar indicates the adjusted *p*‐value. F) The synergy of R4Cl with rifamycin for *M. tuberculosis*, with a calculated FIC.

To verify if supercoiling is affected as anticipated, we used biotinylated trimethyl psoralen (bTMP), which preferentially binds to negatively supercoiled DNA,^[^ ^57^ ^]^ to determine whether CHADs can influence the DNA supercoiling state. We examined two model bacteria, *E. coli* (MG1655) and B. subtilis (168), commonly used in supercoiling studies, to compare their DNA supercoiling levels with and without R4Cl treatment. During the exponential phase, the normalized fluorescence intensities (AU per cell) for *E. coli* and *E. coli* treated with R4Cl were 891 and 456, respectively (**Figure** **7**A). Similarly, the normalized fluorescence intensities for *B. subtilis* and *B. subtilis* treated with R4Cl were 8465 and 3841, respectively (Figure 7B). The decrease in fluorescence intensity indicates a reduction in supercoiling levels, demonstrating the supercoiling relaxation property of R4Cl. As supercoiling is a master transcriptional regulator for bacteria, the relaxed supercoiling level would affect global gene expression.^[^ ^58^ ^]^ Indeed, transcriptome analysis (KEGG enrichment) revealed an enrichment of stress‐response genes in the presence of R4Cl, despite the compound not inhibiting *E. coli* growth at 10 µg mL^−1^ or causing morphological changes (Figure S15A–C, Supporting Information).

Since dynamic supercoiling adjustments are crucial for bacterial adaptation to environmental stresses, R4Cl‐induced relaxation may impair this regulatory mechanism, preventing bacteria from fine‐tuning their supercoiling in response to changing conditions. We then tested whether the relaxed supercoiling state induced by R4Cl could influence the bacterial response to other antibiotics. Using carbenicillin (CB) as an example, the phenotype of R4Cl‐treated *E. coli* was notably different from the control: *E. coli* cells exhibited a characteristic elongated shape under CB exposure, whereas most R4Cl‐treated cells were significantly shorter under the same conditions (Figure 7C). Furthermore, the MIC of CB decreased from 10 to 8 µg mL^−1^ in the presence of R4Cl. Subsequent transcriptome analysis revealed altered gene expression patterns in the presence of R4Cl (Figure 7D,E). This alteration closely resembles the transcriptional profile of *E. coli* when its HU regulatory system is deleted, characterized by the absence of secondary metabolite biosynthesis and the enrichment of the two‐component system.^[^ ^59^ ^]^ Secondary metabolites are auxiliary compounds that are not essential for normal cell growth but crucial for environmental adaptation,^[^ ^60^ ^]^ while the two‐component system also enables bacteria to sense and respond to environmental changes.^[^ ^61^ ^]^ Additionally, metabolic adaptation to diverse environments was the most enriched pathway in control cells but was notably absent in R4Cl‐treated cells. These results show that while bacteria can partially compensate for the loss of supercoiling regulation through the two‐component system, this compensation appears to be incomplete, as evidenced by the drop in MIC for CB. Thus, these findings further support that R4Cl can misdirect the optimal bacterial responses to CB by functioning as a HU inhibitor.

Given the established role of supercoiling in bacterial antibiotic resistance,^[^ ^62^ ^]^ we therefore tested the potential antibiotic synergy effect of R4Cl with rifamycin, as antibiotic combinations have become standard therapy for treating tuberculosis.^[^ ^63^ ^]^ The checkerboard assay, based on the resazurin microtiter plate method, clearly demonstrates synergy between R4Cl and rifamycin. The fractional inhibitory concentration (FIC) index was calculated to be 0.21, indicating a strong interaction between the two drugs (Figure 7F). Thus, R4Cl could serve as an adjuvant agent as part of combination therapies for tuberculosis due to its synergistic effect with rifamycin.

## 3 Discussion

The application of phages has been extensively explored to compensate for the current situation of antibiotics and tackle multidrug‐resistant bacterial infections.^[^ ^64^, ^65^, ^66^ ^]^ In addition, phage‐inspired target discovery exploits phage hijacking strategies to facilitate the development of novel small‐molecule antibiotics.^[^ ^67^ ^]^ While bacterial RNA polymerase (RNAP) is arguably one of the most common phage targets, the cell division protein FtsZ, DNA replication protein β‐clamp, HU, and glycolysis enzyme enolase are among the diverse targets that phages use to hijack host metabolism.^[^ ^17^, ^68^, ^69^, ^70^ ^]^ In this study, we drew inspiration from phage SPO1 protein Gp46 and selected its interfaces with the bacterial HU protein as sites for virtual drug screening. After identifying the initial compound and its key functional group, we expanded our search to develop a lead compound library. Following validation at molecular, bacterial, and animal model levels, we confirmed the antibacterial activity of CHADs. Since the anti‐cancer drugs like Belinostat and Panobinostat have been proven safe in clinical trials, repurposing them presents a fast‐track and cost‐effective approach. Moreover, R4Cl is a promising candidate for further development, as it has improved effectiveness against clinically significant bacterial pathogens, including *S. aureus, A. baumannii*, and *M. tuberculosis*. Furthermore, Apicomplexans such as T. gondii and P. falciparum also encode essential HU‐like proteins. Therefore, the potential inhibitory effects of CHADs should also be evaluated in these eukaryotic pathogens.

Biofilms provide a protective environment that significantly enhances bacterial survival against antibiotics. Although biofilms exhibit resistance to antibiotic treatment due to multiple tolerance mechanisms, some antibiotics, like macrolides, have anti‐biofilm properties.^[^ ^71^ ^]^ However, CHADs’ mechanism of action differs from that of macrolides, which inhibit the synthesis of the proteins that are responsible for biofilm formation.^[^ ^72^ ^]^ HU has been shown to play a key role in biofilms: it can be released into the extracellular matrix through cell lysis, explosive cell lysis, or secretion via the type IV secretion system, where it acts as a structural scaffold by binding extracellular DNA and other matrix components, thereby stabilizing biofilm architecture.^[^ ^19^ ^]^ By targeting HU, CHADs both prevent biofilm formation and disrupt established biofilms. Notably, the rare Z‐form DNA, which is critical for biofilm structure, can be converted back to B‐form DNA under CHAD treatment. Furthermore, due to the high sequence similarity between IHF and HU, particularly the conservation of key residues required for CHAD binding, CHADs may also act as IHF inhibitors, although this requires further investigation. Taken together, CHADs exhibit potent biofilm eradication activity, making them promising candidates for the treatment of persistent biofilm‐associated infections.

DNA supercoiling occurs naturally during cellular processes such as DNA replication and transcription, and its balance is controlled antagonistically by DNA gyrase and topoisomerase I. Bacterial topoisomerase inhibitors, such as quinolones, constitute a class of antibiotics that target DNA gyrase and topoisomerase IV, inhibiting the DNA breakage and rejoining process essential for DNA/RNA synthesis.^[^ ^73^ ^]^ NAPs, such as HU, also regulate supercoiling through their interactions with DNA. However, unlike topoisomerases, HU does not introduce DNA breaks. Instead, the HU‐DNA interaction is considered a general mechanism for bacterial transcriptional regulation during the cell cycle and stress response.^[^ ^20^ ^]^ CHADs exhibit a strong supercoiling relaxation effect by disrupting the HU‐DNA interaction, thereby interfering with bacterial stress responses to antibiotic exposure. As demonstrated during CB treatment, CHADs altered the expression of genes required for CB tolerance and affected bacterial morphology. Consequently, due to the strong biofilm eradication effect of R4Cl, it is a promising candidate for treating persistent biofilm‐associated infections.

The additional anti‐biofilm and anti‐supercoiling effects make CHADs rather unique candidates for further development as stand‐alone antibiotics. Likely due to the combination of these counteracting effects against bacterial stress responses, CHADs exhibit a low frequency of resistance and a strong synergistic effect when used with other antibiotics. Furthermore, its synergy with rifamycin may provide new therapeutic options for treating tuberculosis, making it a potential adjuvant to enhance the efficacy of other antibiotic treatments.

## 4 Experimental Section

### Virtual Screens

OpenMM^[^ ^74^ ^]^ was used for energy minimization of 2.14 Å SaHU structure (PDB code 4QJN). The coordinate of C_α_ of K80 of site 1 is set as the center of the docking grid box with a size of 20 Å^3^, and for site 2, the coordinate of I71 C_α_ was used. AutoDock vina (v1.2.0)^[^ ^43^ ^]^was used to dock the molecules of both DrugBank^[^ ^75^ ^]^ and ZINC20^[^ ^42^ ^]^ to SaHU. Additionally, CPI scores were also predicted using Pangu Drug Model CPI predictor^[^ ^76^ ^]^ for the molecules from the DrugBank. The geometric means of the docking and CPI scores were used to rank the molecules as previously described.^[^ ^77^ ^]^ All DrugBank molecules are ranked based on their combined scores and listed in Tables S1 and S2 (Supporting Information), with the top 10 moleluces are highlighted in green (Supporting Information). And top 1000 compounds for the ZINC20 are ranked by docking score and listed in Tables S3 and S4 (Supporting Information).

### Cloning, Expression and Purification of the Recombinant HU Proteins

All HU genes were obtained as full‐length DNA fragments through de novo chemical synthesis in pGEM plasmids and subsequently amplified by PCR. And the PCR products were purified by 1.5% agarose gel electrophoresis and ligated into respective linearised expression vector based on Gibson‐assembly, and the cloned plasmids were implied in *E. coli* strain DH5α and verified by sequencing. Site‐directed mutagenesis was performed by Q5 Site Directed Mutagenesis Kit (NEB, Cat#E0552S). All primers and plasmids are shown in Table S7 (Supporting Information).

The vectors were then transformed into BL21(DE3) cells to express recombinant proteins. Bacterial cultures were incubated at 37 °C in LB medium or M9 medium (for isotopic labeling, using ^15^N‐labelled NH_4_Cl and/or ^13^C‐labelled glucose) containing relevant antibiotics, grown to OD_600_ of ≈0.6, and induced by 1 mM isopropyl‐β‐D‐thiogalactopyranoside (IPTG). After shaking at 18 °C for 16–18 h, the cells were harvested by centrifugation at 5000 rpm for 10 min at 4 °C and resuspended in a 30 mL binding buffer (50 mm NaH_2_PO_4_, 300 mm NaCl, 10 mm imidazole, and pH 8.0). The cells were then lysed by sonication and centrifuged at 20 000 rpm at 4 °C for 30 min. The supernatants of cell lysate were loaded onto the Ni‐NTA agarose resin (Qiagen, Cat#30 250) in the presence of binding buffer. The resin was washed with a wash buffer (binding buffer with 20 mm imidazole), and then the recombinant proteins were eluted by a elution buffer (binding buffer with 250 mm imidazole). The eluted fractions were dialyzed against the storage buffer (50 mm NaCl, 20 mm Tris, pH 7.4). As for SUMO‐HU, the elution was also digested with protease Ulp1. The SUMO tag and Ulp1 were removed by the second immobilized metal affinity chromatography. And the HUs were loaded on a 5 mL HiTrap Heparin HP column (GE Healthcare Life Sciences), eluting with a linear gradient of 0.05–1.5 m NaCl. The recombinant proteins were further purified by size exclusion chromatography using a Superdex 200 Hiload 16/600 column (GE Healthcare Life Sciences), which was equilibrated with the storage buffer, on an ÄKTA pure system (GE Healthcare Life Sciences). The concentrations of the proteins were estimated by the Bradford protein assay.

### Water Ligand Observed Gradient Spectroscopy (WaterLOGSY)

The 1D NMR WaterLOGSY experiments of 500 µm compounds were carried out at 298 K using an 800 MHz Avance Neo spectrometer (Bruker) equipped with a cryogenic probe, followed by WaterLOGSY experiments of samples consisting of 10€µm HUs and 400 µm compounds. All samples were prepared in the storage buffer, containing 5% DMSO and 10% D_2_O. The WaterLOGSY (*ephogsygpno*) pulse sequence from Bruker was used for data acquisition with 256 scans.

### Binding Affinity Determination by Microscale Thermophoresis (MST)

Due to the strong non‐specific binding of CHADs to the probes or chips, biolayer interferometry (BLI) or surface plasmon resonance (SPR) did not produce reproducible results for the CHADs‐SaHU interactions. And an equilibrium could not be established in isothermal titration calorimetry (ITC) due to the poor solubility of the CHADs. MST was used for the binding affinity determination. MST was performed to study the interactions between SaHU and the inhibitors. Purified SaHU was labelled with a fluorescent dye using the Protein Labelling Kit RED‐NHS kit (NanoTemper Technologies, CAT#MO‐L011) according to the manufacturer's instructions. Binding assays were performed with a Monolith NT.115 device (NanoTemper Technologies) using standard treated capillaries. Compounds were serially diluted two‐fold in MST buffer (PBS containing 500 mm NaCl and 0.05% Tween‐20, pH 7.4). The concentration of labelled protein was kept to a minimum (20 nm). Equal amounts of labelled protein were titrated with varied ligand concentration (0.24 µm to 0.5 mm). The dissociation constant (K_D_) values were obtained by fitting the binding curve with the NT Analysis software from Nano Temper Technologies.

### Electrophoretic Mobility Shift Assay (EMSA)

The sequences of the oligonucleotides used in EMSA are listed in Table S5 (Supporting Information). The 5′ end of the leading strand was labelled with FAM. The 21 bp dsDNA was prepared by annealing the labelled leading strand to its non‐labelled complementary oligonucleotide. Belinostat was dissolved in DMSO to prepare a stock solution with a concentration of 100 mM. SaHU and the 21 bp dsDNA were pre‐incubated on ice for 30 min. Different concentrations of Belinostat were added to the SaHU‐dsDNA complex, respectively. After adding Belinostat, the mixtures were incubated on ice for 5 min and then loaded onto a 7% (w/v) non‐denaturing polyacrylamide gel. The electrophoresis was performed at 90 V in 0.5 × TBE buffer on ice. The image was captured by a ChemiDoc MP Imaging System (Bio‐Rad).

### Antibiotic Susceptibility Testing (AST) and Minimum Inhibitory Concentration (MIC) Determination

The AST using the disk diffusion method was performed before the MIC determination according to CLSI standards.^[^ ^45^ ^]^ Briefly, each compound was dissolved in DMSO at a moderate. 20 µL of each drug solution was added to a blank disk before use. Disks containing DMSO were used as negative controls. A direct LB broth suspension of *S. aureus* was prepared by isolating colonies from an 18‐ to 24 h LB agar plate and then adjusted to a turbidity equivalent to a 0.5 McFarland standard using a photometric device. Within 15 min after adjusting the turbidity of the inoculum suspension, a sterile cotton swab was dipped into the suspension and then streaked over the entire sterile LB agar surface to ensure even distribution of the inoculum. The lids were ajar for 3–5 min to allow any excess surface moisture to be absorbed before applying the drug‐impregnated disks. The plates were incubated at 37 °C for 24 h. The images were captured by a ChemiDoc MP Imaging System (Bio‐Rad).

For the MIC determination, 100 µL of the mixtures containing bacterial suspensions and the small molecules to the required final concentration in 96‐well flat‐bottom plates. The final cell density of each well was ≈5 × 10^5^ CFU mL^−1^. Plates were then incubated at 37 °C without shaking overnight until untreated control cultures reached stationary phase. Then plates were read at 600 nm using a Cytation 5 plate reader (BioTeK). All MICs expect for *M. tuberculosis* were determined at the BioBank, the first affiliated hospital of Xi'an Jiaotong University (Biosafety level 2). The MICs for *M. tuberculosis* and the XDR‐TB strain were detected by resazurin microtitre plate assay in Henan Center for Disease Control and Prevention (Biosafety level 3). Bacterial suspension equivalent in turbidity to that of 1 McFarland standard was diluted 1:200 in Middlebrook 7H9 broth. 200 µL inoculum and various concentrations of R4Cl were added in each well of a 96‐well plate. Plates were sealed and incubated at 37 °C for 2 weeks. Resazurin solution (0.02%) was added to the plates (25 µL/well) and incubated at 37 °C for 24 h. The MIC was determined as the corresponding solution showing a blue color. All experiments containing at least three technical replicates were repeated at least twice. All bacterial strains used are listed Table S6 (Supporting Information). Quality control was performed for each bacterium using tobramycin for Gram‐positive bacteria (except for *M. tuberculosis*), erythromycin for Gram‐negative bacteria, and rifamycin for *M. tuberculosis* (Figure S13B, Supporting Information).

### Gram Staining and Transmission Electron Microscopy (TEM)

*B. subtilis* (strain 168), *S. aureus*, and *E. coli* were grown at 37 °C overnight in 3 mL of LB medium and diluted 1:100 (v/v) into fresh LB medium. *B. subtilis* (strain 168) and *S. aureus* were treated with or without 400 µg mL^−1^ (1.26 mm) of Belinostat for another 4 h, while *E. coli* was exposed to 10 µg mL^−1^ of CB, 10 µg mL^−1^ of R4Cl or 10 µg mL^−1^ of CB and 10 µg mL^−1^ of R4Cl. The Gp46‐overexpressed *B. subtilis* strain was prepared as described previously.^[^ ^78^ ^]^

For Gram staining, bacteria were harvested by centrifugation at 4 000 rpm for 10 min and washed twice with PBS buffer. The cells were resuspended in PBS, and 5 µL of the bacterial suspension was spread onto clean glass slides to create thin films. The slides were passed through a flame 3 to 4 times to heat‐fix the bacteria to the slide. For Gram staining, the slides were strained first with crystal violet solution for 1 min followed by Gram's iodine solution for another 1 min. 95% ethanol was used to decolorize the slides, and the safranin solution was used as counterstain. The slides were air‐dried and covered with cover slips. Images were taken with a CX23 upright microscope (Olympus).

For the TEM, the samples were prepared as described.^[^ ^68^ ^]^ The prepared sample sections were

examined with a JEM‐1400 FLASH Transmission Electron Microscope (JEOL). Three technical replicates were performed to ensure the consistency of the observation.

### Lead Compound Library Expansion

The compound expansion was performed online using PGMO, part of Pangu Drug Model.^[^ ^76^ ^]^ 1820 molecules were generated and ranked by the Quantitative Estimate of Druglikeness (QED) were listed in Table S7 (Supporting Information).

### X‐Ray Crystallography

The SaHU protein was diluted to 8 mg/mL for crystallization. Crystals grew with hanging drops at 16 °C in crystallization buffer (0.1 m MES pH 6.0, 15% w/v PEG 3350, 20% glycerol). The crystals were mounted and flash‐frozen in liquid nitrogen. Diffraction data were collected at the Shanghai Synchrotron Radiation Facility beamline BL02U1, and a total of 360 frames were recorded with 1° oscillation. For structure determination and analysis, HKL2000 was used to index, integrate, and scale the data.^[^ ^79^ ^]^ Molecular replacement was performed with PHASER using the search model of SHU (PDB ID: 4QJN). The final model was built with COOT,^[^ ^80^ ^]^ and refined in PHENIX.^[^ ^81^ ^]^ The model quality was analyzed by Molprobity,^[^ ^82^ ^]^ and deposited in the Protein Data Bank with accession code 8HD5. Statistics from data collection, processing, and structure refinement are summarized in Table S8 (Supporting Information).

### NMR Backbone Assignment and Titration Experiment

The triple resonance experiments were performed in a buffer containing 500 mm NaCl, 50 mm KH_2_PO_4_, 10% D_2_O, and pH 7.4. NMR spectra were collected at 298 K using an 800 MHz Avance Neo spectrometer (Bruker) equipped with a cryogenic probe. Spectral assignments were completed using our in‐house, semi‐automated assignment algorithms and standard triple‐resonance assignment methodology.

The NMR titration experiments were performed in the storage buffer containing 5% DMSO and 10% D_2_O. ^1^H‐^15^N HSQC spectra were acquired at 298 K for each sample, and the intensity change (I/I_0_) were calculated, where I and I_0_ correspond to the peak intensity of the bound and free SaHU^D15G^, respectively.

### HADDOCK Model

The exposed residues of SaHU (as shown in Figure 3D) that experienced major peak broadening effects, i.e., L29, D40, T43, L44, I45, E51, K80, A81, and K83 were chosen as the active residues, and the surrounding residues were designated as passive residues. And all atoms of R4Cl were used to build a model of a 2:2 complex with one R4Cl bound to one subunit of SaHU^D15G^ using HADDOCK (v2.2).^[^ ^52^ ^]^ An ambiguous distance restraint of 2.0 Å was invoked between all active and passive residues of the other protein partner. The interfacial residues were allowed to move during the simulated annealing and water refinement. A total of 1000 initial complex structures were generated by rigid‐body energy minimization, and the best 200 by total energy were selected for torsion angle dynamics and subsequent Cartesian dynamics in an explicit water solvent. Structures were ranked based on interaction energy, resulting in five clusters. The best cluster, selected as the final model, showed the lowest energy with a HADDOCK score of −147.3 ± 5.6 and a Z‐score of −2.5. In contrast, the second‐best cluster exhibited markedly weaker binding, with a HADDOCK score of −47.6 ± 4.4 and a Z‐score of −0.7.

### Molecular Dynamic(MD) Simulation

The MD simulation of the CHAD‐SaHU^D15G^ complex was performed using the Molecular Mechanics Poisson–Boltzmann Surface Area (MM‐PBSA) method to evaluate the protein–ligand interaction.^[^ ^83^ ^]^ The MMPBSA simulation was implemented through package SPONGE^[^ ^84^ ^]^ and the protein‐ligand binding free energy and estimation of the energy contribution per residue to the binding were calculated using *MMPBSA.py*.^[^ ^85^ ^]^ The topology of the protein is constructed using ff99sb force field, and the ligand topologies were generated from the gaff force field with bcc charges. The water model (tip3p) was used to solvate the system in a dodecahedron unit cell. The resultant fully solvated system was then neutralized with sodium ion. SPONGE was used to generation a 2 ns production phase MD simulation (Video S1, Supporting Information). The free energy calculation based on PBSA model and the energy decomposition to each residue were performed by AmberTools22.^[^ ^86^ ^]^

### Relative Binding Free Energy (RBFE) Calculation

The RBFE calculations were performed using our in‐house SPOGNE‐FEP based on SPOGNE, a GPU‐accelerated molecular dynamics package that uses enhanced sampling and AI‐driven algorithms to improve accuracy.^[^ ^84^ ^]^ Belinostat as the reference compound and R4Cl‐SaHU model were used as the complex structure in the RBFE calculation for all MIC‐determined molecules listed in Figure 2. The calculations were performed on a Nvidia V100 GPU computer and took ≈4 h for one pair of molecules.

### Minimum Bactericidal Concentration (MBC) Determination

The cidality assay was carried out according to CLSI M26‐A guideline. Briefly, an exponential culture of *S. aureus* was washed then diluted to 5–7.5 × 10^5^ CFU mL^−1^ in 2 mL of LB medium, supplemented with 0 (growth control), 2, 4, 6, 8, 16, and 32 times the MIC of R4Cl. The samples were incubated at 37 °C without shaking. The final inoculum was confirmed by actual count. Tubes were vortexed and re‐incubated at 20 h, then vortexed and re‐incubated again before sampling at 24 h. Aliquots of the culture (0.1 mL) were diluted once in antibiotic‐free LB. Both 0.1 mL of the culture and 0.1 mL of the dilution were streaked onto agar plates separately. The plates were incubated at 37 °C for 24 h. Colonies were then counted, with a **limit of detection of 100 CFU/mL**. The lethal endpoint was defined as 99.9% killing (≥ 3 log_10_ drop in CFU/mL) of the final inoculum.

### Frequency of Resistance (FoR)

Log‐phase *S. aureus* at concentrations ranging from 10^6^ to 10^10^ CFU mL^−1^ were plated onto LB agar plates containing R4Cl at eight times of its MIC. The plates were incubated at 37 °C for 48 h. Additionally, dilutions of each culture were plated on drug‐free LB agar to ensure accurate colony counts. Resistance frequencies were calculated by dividing the number of resistant colonies by the total number of CFU plated. Each experiment was performed in triplicate, and the reported frequency represents the average value.

### Genome Sequence Analysis

Sourmash was used to compute the sourmash signature for every bacterial assembled genome and produce the similarity heatmap as previous described.^[^ ^87^ ^]^ The collection of the clinical isolates was approved by the Committee of Ethics (Approval NO. 2022‐1120).

### Toxicity Assay of CHADs in Mice

All experimental procedures were conducted in accordance with animal welfare guidelines and were previously approved by the Animal Welfare and Ethics Committee of Xi'an Jiaotong University, Xi'an, China (Approval No. 2021‐1513). In these studies, 6‐ to 7‐week‐old female specific pathogen‐free (SPF) Kunming mice purchased from the Laboratory Animal Center of Xi'an Jiaotong University (Xi'an, China) were used. Animals were housed under standard conditions of humidity (50% ± 10%), temperature (22 ± 2 °C), and light‐dark cycle (12 h each) with free access to food and water.

To assess the percutaneous toxicity of CHADs in mice, the mice were orally administered 45 µL of Ibuprofen suspension 1 h before being anesthetized with isofluorane. An exposed skin area (2 cm × 1 cm) was created by depilating the back skin of the mice using hair removal cream. Kunming mice were randomly divided into four groups (*n* = 6) and weighed once a day. Considering the low solubility, CHADs were dissolved in 10% DMSO and 90% PEG 400. A dose of 1000 mg kg^−1^ of Belinostat, Panobinostat or R4Cl was applied to medical gauze. The medical gauze was placed snugly against the exposed skin of the mice and secured with medical tape to ensure it did not interfere with the normal activities of the mice. The gauze was removed after 4 h. The exposed skin of the mice was gently wiped with a cotton ball dampened with warm water. These steps were repeated for seven consecutive days, and the health conditions of the mice were observed daily. The mice were euthanized and dissected seven days later, and the heart, liver, spleen, lung, and kidneys were collected for further analysis.

Belinostat, Panobinostat, and R4Cl were formulated in 10% DMSO, 40% PEG 400, 5% Tween‐80, and 45% saline. For the acute toxicity assay, the LD50 of a single compound was determined via intravenous injection using the up‐and‐down procedure.^[^ ^53^ ^]^ The estimated initial LD50 was 175 mg kg^−1^. Sigma was 0.2, the slope was 5, and T was 1.6. The sequential dosages were calculated using AOT425‐StatPgm. The first dosage of 175 mg kg^−1^ was administered to the first mouse. If it survived within 24 h, 280 mg kg^−1^ was given as the second dose according to the sequential dosages. If it died, the mouse was dissected, and 110 mg kg^−1^ was chosen. Tests continued until the standard stopping criteria were met. All surviving mice were monitored for signs of toxicity and then euthanized and dissected 14 days later. For the multiple‐dose assay, CHADs were administered daily via intravenous injection for seven consecutive days. Belinostat and R4Cl were given at a daily dose of 44 mg kg^−1^, and Panobinostat was given at 28 mg kg^−1^ (*n* = 6 for each compound). Control mice received the same volume of the solvent. The mice were monitored for signs of toxicity, weighed daily, and then euthanized and dissected 7 days later.

Mice were anesthetized with isoflurane, and whole blood was collected from the retro‐orbital venous plexus using a 1 mg mL^−1^ EDTA solution as an anticoagulant. After blood collection, the samples were gently inverted several times to mix well and then stored at 4 °C. Blood cells and platelets were analyzed using a blood analyzer (Kangte). After decapitation, the tissues were promptly fixed in 4% paraformaldehyde for 24 h. The organs were then washed and dehydrated through a series of graded ethanol baths before being embedded in paraffin wax. Serial sections, each 5 µm thick, were cut using a microtome, stained with H&E, and examined using a NanoZoomer S60 (HAMAMATSU).

### Mouse Superficial Skin Infection and Sepsis Model

The animal tests were adapted from a previously described skin infection model in mice^[^ ^88^ ^]^ and performed at BioBank, the first affiliated hospital of Xi'an Jiaotong University, with approval from the Committee of Animal Ethics (Approval NO. 2021‐1513). The 6‐ to 7‐week‐old female specific pathogen‐free (SPF) BALB/c mice were used for the experiments, with *n* = 6 for every group. First, the mice were orally administered 45 µL of Ibuprofen suspension 1 h before being anesthetized with isofluorane. An exposed skin area (2 cm × 1 cm) was created by depilating the back skin of the mice using hair removal cream. Afterward, medical tape was used by the same operator to attach to the depilated skin, and it was avulsed 20 times after applying the same pressure by pressing with their fingers. The medical tape was replaced in each paste‐avulsion process for better removal of the stratum corneum. *S. aureus* and FA‐resistant MRSA with ≈10^7^ CFU mL^−1^, were immediately applied to the skin of each animal using 20 µL bacterial suspension. After inoculation for 24 h, a group of mice were euthanized, and the 2 cm^2^ infected wound skin was promptly removed through surgery to quantify the infectious dose before any treatment was given. The skin was immediately serially diluted in saline, crushed, and ground using a tissue homogenizer. The suspensions were diluted with saline, and the dilutions were smeared on LB agar plates. These plates were incubated at 37 °C for 24 h. The colonies were counted and converted to the bacterial content per gram of tissue (CFU/g). All small molecules were diluted from a 100 mm DMSO stock solution using 90% saline containing 10% PEG 400 to the desired concentration. The remaining mice were treated with 100 µL of 90% saline containing 10% PEG 400 (control), small molecule solutions, and FA (2%), applied to the skin lesions twice daily (9 a.m. and 9 p.m.) for three consecutive days. The food intake, drinking water, and mental status of the mice were observed at least twice a day. The mice did not show any signs of systemic infection, weight loss, or distress. The mice were then euthanized on day 4, 12 h after the last treatment. The 2 cm^2^ infected wound skin was promptly removed through surgery. Half of this excised tissue was embedded in paraffin for subsequent histological examination using H&E staining, and the other half of the skin was immediately serially diluted in a saline solution, crushed, and ground using a tissue homogenizer. The bacterial content per gram of tissue (CFU/g) was detected as mentioned before.

For the sepsis model, 8‐week‐old female BALB/c mice were used, with *n* = 10 for each group. The mice were injected in the tail vein with lethal doses of *S. aureus* (1 × 10^8^ CFU) or *P. aeruginosa* (8 × 10^8^ CFU) as previously described to cause septic shock.^[^ ^89^, ^90^ ^]^ Belinostat and R4Cl were formulated in 10% DMSO, 40% PEG 400, 5% Tween‐80, and 45% saline. The solvent (control), 40 mg kg^−1^ of Belinostat or 3 mg kg^−1^ R4Cl was injected intravenously after 5 h. The mice were monitored for 48 h after the drug injection, and the remaining mice were sacrificed on day 3. Statistical analysis of Kaplan‐Meier survival curves of each group of mice was performed using the log‐rank (Mantel‐Cox) test.

### Mass Spectroscopy Analysis of HU Population in Different Forms

*S. aureus* was initially cultured in BHI broth at 37 °C for 18 h. 300 µL of the suspensions were diluted 1:100 (v/v) with BHI broth containing 1% glucose in 50 mL conical sterile polypropylene centrifuge tubes. 15 µg mL^−1^ (0.5 × MIC, 76 µm) of R4Cl was added, and DMSO‐treated *S. aureus* was used as a control. The samples were incubated statically at 37 °C for 24 h. Then the cultures were vortexed, and the microbial counts were detected by plate count. The extraction of EPS was performed according to *Akio Chiba*. et al.^[^ ^91^ ^]^ Briefly, these cultures were centrifuged at 8000 g for 10 min, and the supernatants (growth medium) were concentrated to 500 µL using 10 kDa ultrafiltration tubes. The sediments were resuspended with 500 µL of 1.5 m NaCl solution and centrifuged at 8000 g for 10 min again. The supernatants contained biofilms. The bacteria were resuspended with 500 µL of PBS with 8 m Urea added, lysed by sonication, and centrifuged at 20 000 rpm at 4 °C for 10 min. The supernatants contained cellular material.

5 µL of 5 × loading buffer was added to 20 µL of each supernatant. 10 µL of each sample was loaded into each lane of 20% polyacrylamide gels in the Mini Trans‐Blot Cell system (Bio‐Rad) running at 210 V for 1 h. Gels were stained with Coomassie brilliant blue for no less than 10 min followed by destaining in water overnight. The images were captured by a ChemiDoc MP Imaging System (Bio‐Rad). Sample lanes recycled from the SDS gel were digested with trypsin and desalted by C18 ZipTips (Millipore, Cat# ZTC18S096). The samples were analyzed by a Q‐Exactive plus mass spectrometer (Thermo Fisher Scientific) coupled with an UltiMate 3000 RSLCnano system. Database search was performed by MaxQuant^[^ ^92^ ^]^ and results is shown in Table S9 (Supporting Information).

### Biofilm Formation Inhibition and Eradication Assays

Crystal violet assay was utilized for both the biofilm formation inhibition and eradication assays. The bacteria were initially cultured in BHI broth at 37 °C for 18 h. The suspensions were then adjusted to 0.5 McFarland standard. Subsequently, the suspensions were diluted 1:100 (v/v) with BHI broth containing 1% glucose to obtain suspensions containing 1 × 10^6^ CFU mL^−1^ bacteria.

For the biofilm formation inhibition assay, standardized *P. aeruginosa* suspension at 10^6^ CFU mL^−1^ was dispensed into each well of a 96‐well plate. Various concentrations of R4Cl (0.625, 1.25, 2.5, 5, 10, and 20 µg mL^−1^) were added to the bacterial suspension for the dose‐dependent anti‐biofilm assay. DMSO replaced the compound as a control, and uninoculated broth served as the blank. The plate was then incubated at 37 °C for 24 h under static conditions. After incubation, the wells were washed with PBS buffer, heat‐fixed, and stained with filtered 0.5% crystal violet for 5 min at room temperature. Excess stain was removed by rinsing with water, and bound crystal violet from the cells or biofilms was solubilized with 33% acetic acid. The released stain was measured at 570 nm using a Cytation 5 plate reader (BioTeK). Each assay was conducted with three technical replicates and repeated three times. The figure presented shows one representative experiment.

For the eradication assay, *P. aeruginosa* and *S. aureus* were initially cultured in BHI broth at 37 °C for 18 h. Standardized suspensions containing 10^6^ CFU mL^−1^ bacteria were incubated in 96‐well microtiter plates at 37 °C for 24 h to allow biofilm formation. Afterward, the media were removed, wells were rinsed with PBS, and various concentrations of R4Cl (10, 20, 30, 40, 50, and 60 µg mL^−1^) were added and incubated for another 24 h. The wells were emptied, heat‐fixed, stained with 0.5% crystal violet, rinsed, air‐dried, and 33% acetic acid was added for absorbance measurement at 570 nm. DMSO replaced R4Cl as the negative control. Each assay was performed with three technical replicates and repeated three times. The figure presented shows one representative experiment.

The amount of biofilm was calculated according to the following formula (where OD indicates optical density):

(1) $$\begin{array}{ccc} \mathrm{Relative}\;\mathrm{biofilm}\;\mathrm{density}\left(\%\right) & = & OD_{570\mathrm{sample}}/\mathrm{average}\;\mathrm{of}\;OD_{570\mathrm{negative}\mathrm{control}}\\ & & \times \;100\% \end{array}$$

The biofilm conditions were observed by SEM. For the biofilm formation inhibition assay, sterile polystyrene coverlips (1 mm thick and 5 mm in diameter) were placed into the wells of 6‐well plates for biofilm formation. 2 mL of standardized *P. aeruginosa* suspension at 10^6^ CFU mL^−1^ was dispensed into each well. 20 µg mL^−1^ of R4Cl was added. The polystyrene coverlips with cells grown in R4Cl‐free medium were utilized as controls. The coverlips were incubated at 37 °C for 24 h and then gently washed three times with PBS to remove non‐adherent bacteria. The adherent bacteria were fixed and dehydrated. After being fixed with 2.5% glutaraldehyde for 3 h at 4 °C, the surfaces were rinsed three times and subsequently fixed with 0.1% osmium tetraoxide for 1 h. The samples were dehydrated through a graded ethanol series (50%, 70%, 80%, 90%, 95%, and 99.5%) for 10 min each at room temperature. After critical‐point drying and 1200 bar pres‐sure at 40 °C, the samples were examined using an XL‐30 ESEM (Philips). For the eradication assay, the sterile polystyrene coverlips were placed into the wells of 6‐well plates, too. 2 mL of standardized *P. aeruginosa* and *S. aureus* at 10^6^ CFU mL^−1^ was dispensed into each well and incubated at 37 °C for 24 h to form biofilms. Then, the media were removed, wells were rinsed with PBS, and 60 µg mL^−1^ of R4Cl were added and incubated for another 24 h. The rest steps for the preparation of SEM samples are the same as those mentioned before.

### Quantification of Z‐DNA and B‐DNA Within R4Cl‐Treated Biofilms

Biofilms were allowed to form in a 96‐well glass bottom plate (Cellvis, P96‐0‐N). 200 µL of standardized *P. aeruginosa* or and *S. aureus* suspension at 10^6^ CFU mL^−1^ was dispensed into each well at 37 °C for 72 h. Afterward, biofilms were washed once with PBS, and 20 µg/mL of R4Cl was added. DMSO replaced R4Cl as the negative control. The plate was incubated for another 24 h. The biofilms were then washed with PBS and incubated with 200 µL of PBS that contained 5% (w/v) BSA and either murine monoclonal antibody raised against B‐DNA (Abcam, ab27156) [5 µg mL^−1^] or murine monoclonal antibody against Z‐DNA[Z22] (Absolute Antibodies, Ab00783‐3.0) [5 µg mL^−1^] and their respective murine isotype IgG2a (Invitrogen, 02‐6200) or IgG2b (Invitrogen, 02‐6300) [5 µg mL^−1^] controls for 1 h at room temperature. The biofilms were then washed once with PBS and incubated with the goat‐α mouse IgG conjugated Alexa Fluor405 (Invitrogen, A31553) in PBS that contained 5% (w/v) BSA for 1 h at room temperature, and counterstained with 5 µg mL^−1^ of FM4‐64 (Invitrogen, T3166) in PBS. The biofilms were then washed with PBS and imaged using 60× oil objective on an Olympus FV3000 confocal microscope. The fluorescence intensity of B‐DNA and Z‐DNA was normalized to the fluorescence intensity of FM4‐64. Changes in fluorescence intensity were determined by ImageJ software by determining the mean intensity of Z stacks.

### Biotinylated Trimethyl Psoralen (bTMP) Incorporation and Imaging

*B. subtilis* (strain 168) and *E. coli* were grown at 37 °C overnight in 3 mL of LB medium and diluted 1:100 (v/v) into fresh LB medium. Bacteria were treated with or without 10 µg mL^−1^ of R4Cl for another 4 h.

Bacterial cells were washed with PBS before they were permeabilized with 250 µg mL^−1^ lysozyme for 30 min at room temperature (RT). Cells were incubated with 0.5 mg mL^−1^ EZ‐Link Psoralen‐PEG3‐Biotin (ThermoFisher Scientific) for 20 min, then exposed to 4 kJ m^−2^ of 365 nm light using an UV Crosslinker (SGLinker UV Crosslinker) for 20 min on ice followed by a PBS wash. Cells were then fixed with 4% paraformaldehyde and treated with 0.2% Triton X‐100 before they were incubated with Alexa Fluor 594 Streptavidin (ThermoFisher Scientific) for 1 h at RT in the dark, washed with PBS, and stained with DAPI for 10 min. Cells were then mounted onto glass slides with ProLong Gold antifade mountant (ThermoFisher Scientific). Images were taken with Olympus FV3000 confocal microscope (Olympus). Fluorescence intensity was quantified using ImageJ software from multiple random fields obtained with identical acquisition settings and normalized by cell number.

### Transcription Profiling

For RNA‐Seq, *E. coli* was first grown overnight in LB medium, and then the overnight culture was diluted 1:100 in fresh regular LB medium, LB with 10 µg mL^−1^ of CB, LB with 10 µg/mL of R4Cl or LB with 10 µg mL^−1^ of CB and 10 µg mL^−1^ of R4Cl. Cells were incubated at 37 °C for another 4 h. Three biological replicates per sample were collected for each condition. Total RNA was isolated from each samples using RNeasy Mini Kit (QIAGEN, Cat#74104), and ribosomal RNA was removed with Ribo‐Zero Plus rRNA Depletion Kit (Illumina, Cat#20040529). The remaining RNA was fragmented, converted to cDNA (with dUTP incorporation for strand specificity), and used to create a sequencing library. Library quality was assessed with qBit, Agilent Bioanalyzer, and qPCR. Sequencing was performed on a PE 150 system (Illumina). Reads were mapped to the *E. coli* MG1655 reference genome using Bowtie2, and gene expression was quantified using FeatureCounts (normalized to RPKM). Differential gene expression was determined with DESeq2 (fold change > 1, adjusted *p* < 0.05). KEGG pathway enrichment analysis was performed using clusterProfiler. The RNA‐seq data are available in the SRA under accession number: PRJNA1227296.

### Growth Curve

The bacterial growth assays were performed at 37 °C in 96‐well plates. Briefly, seed cultures were first cultured in 4000 µL of LB medium at 37 °C, shaking at 300 rpm for 16–18 h in an incubator. The cultures were then diluted 1:100 in a final volume of 100 µL of fresh medium with or without 10 µg mL^−1^ of R4Cl. The absorbance at 600 nm was measured at the indicated time points using a Neo 2 multi‐well plate reader (BioTek). At least three biological and technical replicates were performed for each growth curve test.

### In Vitro Synergy Between R4Cl and Rifamycin

The checkerboard method, based on Resazurin microtitre plate assay as described above, was used to study in vitro synergy between R4Cl and Rifamycin. Rifamycin was added at concentrations between 0 and 0.5 µg mL^−1^, and R4Cl was added at concentrations ranging from 0 to 12 µg mL^−1^, as demonstrated in Figure 7F. The FIC was determined using the formula: (MIC of Rifamycin in combination / MIC of Rifamycin alone) + (MIC of R4Cl in combination / MIC of R4Cl alone). Synergy was defined as an FIC < 0.5, indifference as an FIC between 0.5 and 4, and antagonism as an FIC > 4.^[^ ^93^ ^]^

### Statistical Analysis

Data are presented as mean ± SD or SEM, as indicated in the figure legends. Statistical analyses were carried out with GraphPad Prism 9. Statistical comparisons between two groups were performed using an unpaired Student's *t*‐test. A one‐way ANOVA with an alpha of 0.05 is used to compare the means of three or more independent groups to determine if there is a statistically significant difference between them, followed by Dunnett's post‐hoc test. Ns, not significant; ^*^ *p* < 0.05; ^**^ *p* < 0.01; ^***^ *p* < 0.001; ^****^ *p* < 0.0001.

*List of abbreviations*: A complete list of abbreviations used in this study is provided in Table S10 (Supporting Information).

## Conflict of Interest

Bing Liu has filed patent applications for using CHADs in treating infectious diseases. Other authors have no conflicts of interest regarding the work.

## Author Contributions

B.L. conceived the study and designed experiments; H.C. and H.W. prepared the samples, performed the docking, biochemical, cellular, and animal experiments; Y.X. performed NMR analysis; Z.X. performed the AI work and molecular dynamics; X.W. performed the work on Belinostat; Y.K. performed the animal work; Z.W. determined the MICs; Y.L. performed the crystallography‐related work and structure‐based inhibitor screening; W.C. purified the HU proteins and performed WaterLOGSY experiments; M.L. performed full genome sequencing and analyzed the data. X.Z. contributed the clinically isolated bacterial strains. B.L. analyzed the data from all authors and wrote the first draft; B.L. revised the manuscript with input from all authors. H.C., Y.X., Z.X., and H.W. contributed equally to this work.

## Supporting information



Supporting Information

Supplemental Video 1

Supplemental Data

## Data Availability

The structural coordinate of SaHU has been deposited in Protein Data Bank under accession code 8HD5. The sequencing data for the 50 MRSA strains are available under BioProject number SUB14217833. The RNA‐seq data are available in the SRA under accession number: PRJNA1227296.
