# Supplementary material for: Cinnamic‐Hydroxamic‐Acid Derivatives Exhibit Antibiotic, Anti‐Biofilm, and Supercoiling Relaxation Properties by Targeting Bacterial Nucleoid‐Associated Protein HU
Source: Adv Sci (Weinh). 2025 Nov 21;13(13):e09876. doi: 10.1002/advs.202509876 (PMC12955902; doi:10.1002/advs.202509876)
Supplement: Supplementary file 1 — Supporting Information [file ADVS-13-e09876-s002.pdf]

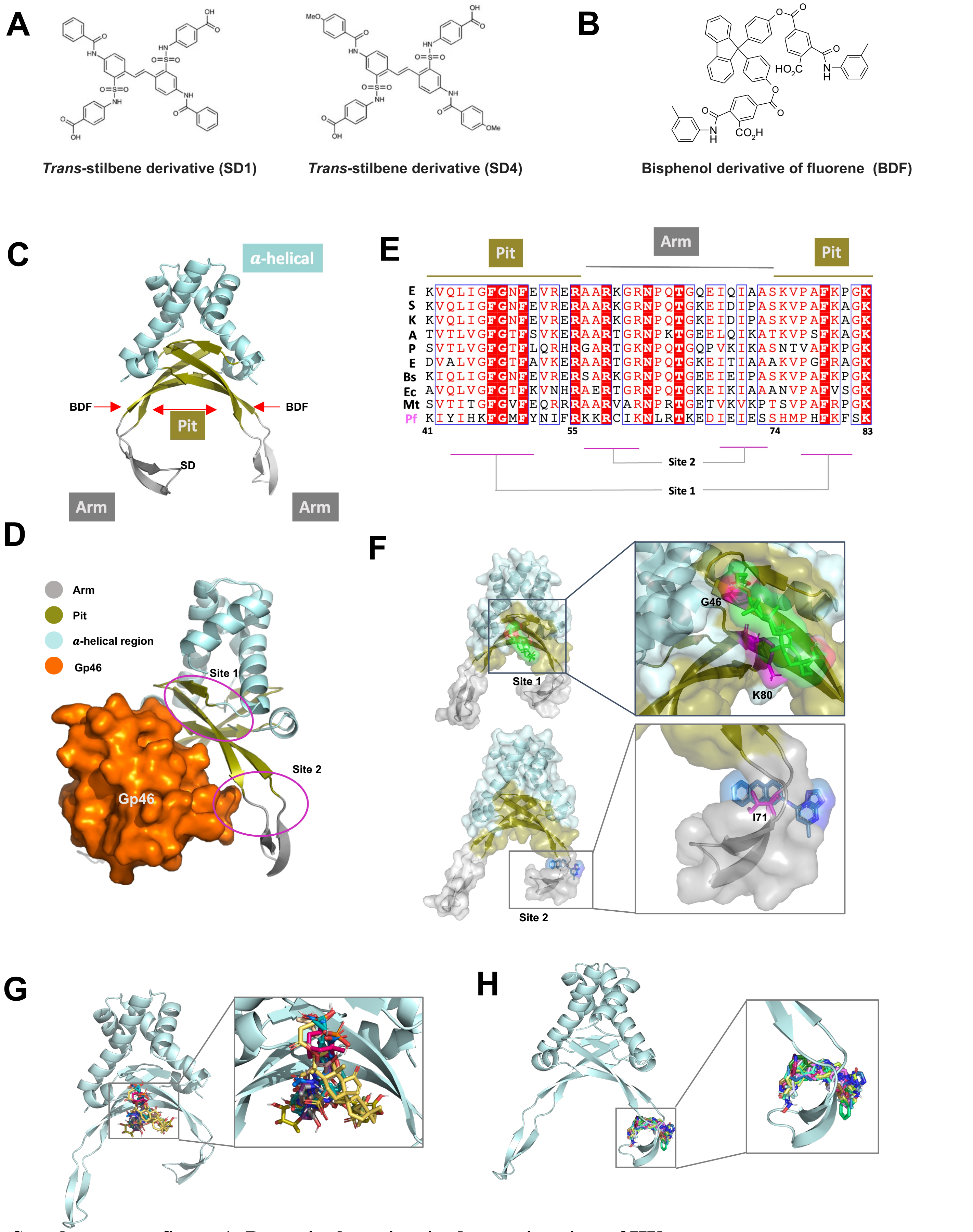

Supplementary figure 1: Bacteriophage-inspired targeting sites of HU.

(A-B) The structural formulas of SD1, SD4 and BDF. **(C)** The proposed targeting sites for BDFs and SDs indicated by the red arrows. The arms, pit and the α-helical regions of Apo SaHU (PDB ID: 4QJN) are colored in silver, olive and cyan, respectively. **(D)** The Gp46-HBsu complex with interface 1 (site 1) and interface 2 (site 2) indicated by circles. **(E)** Multiple sequence alignment of representative bacteria (ESKAPE pathogens), *B. subtilis* (Bs), *E. coli* (Ec), *M. tuberculosis* (Mt), and the eukaryotic parasite *P. falciparum* (Pf) generated using Clustal Omega. Alpha subunit was used if HU is heterodimer. Conserved residues are highlighted, illustrating regions of evolutionary conservation across prokaryotic and eukaryotic sequences. Number is amino acid sequence of Bs. **(F)** Two representative molecules docked on site 1 and 2, using the sites shown in **(D)** and **(E)**. The residues that are key for the interactions are highlighted in purple and numbered. **(G-H)** The cartoon illustration of top 10 drugs from DrugBank for site 1 **(G)** and site 2 **(H)**.

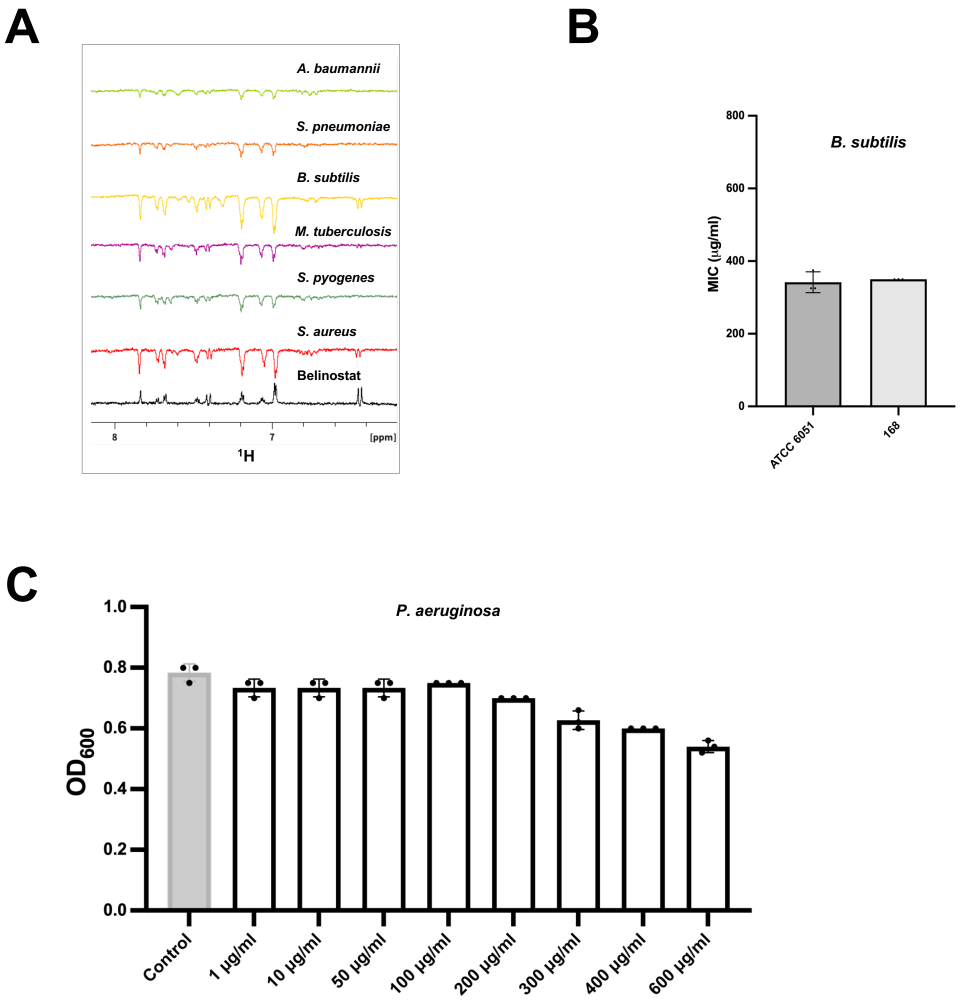

Supplementary figure 2: Belinostat interacts with HUs of different bacteria and is a growth inhibitor in *B. subtilis*.

(A) 1D NMR WaterLOGSY spectra of Belinostat (black) and Belinostat with HUs from *A. baumannii* (lime), *S. pneumoniae* (orange), *B. subtilis* (yellow), *M. tuberculosis* (purple), *S. pyogenes* (green) and *S. aureus* (red). **(B)** MICs of Belinostat on *B. subtilis* strains (ATCC 6051 and 168). Plots show three technical replicates (n = 3), and error bars represent the SEM. **(C)** Belinostat has no inhibitory effect on *P. aeruginosa.* Plots show three technical replicates (n = 3), and error bars represent the SEM.

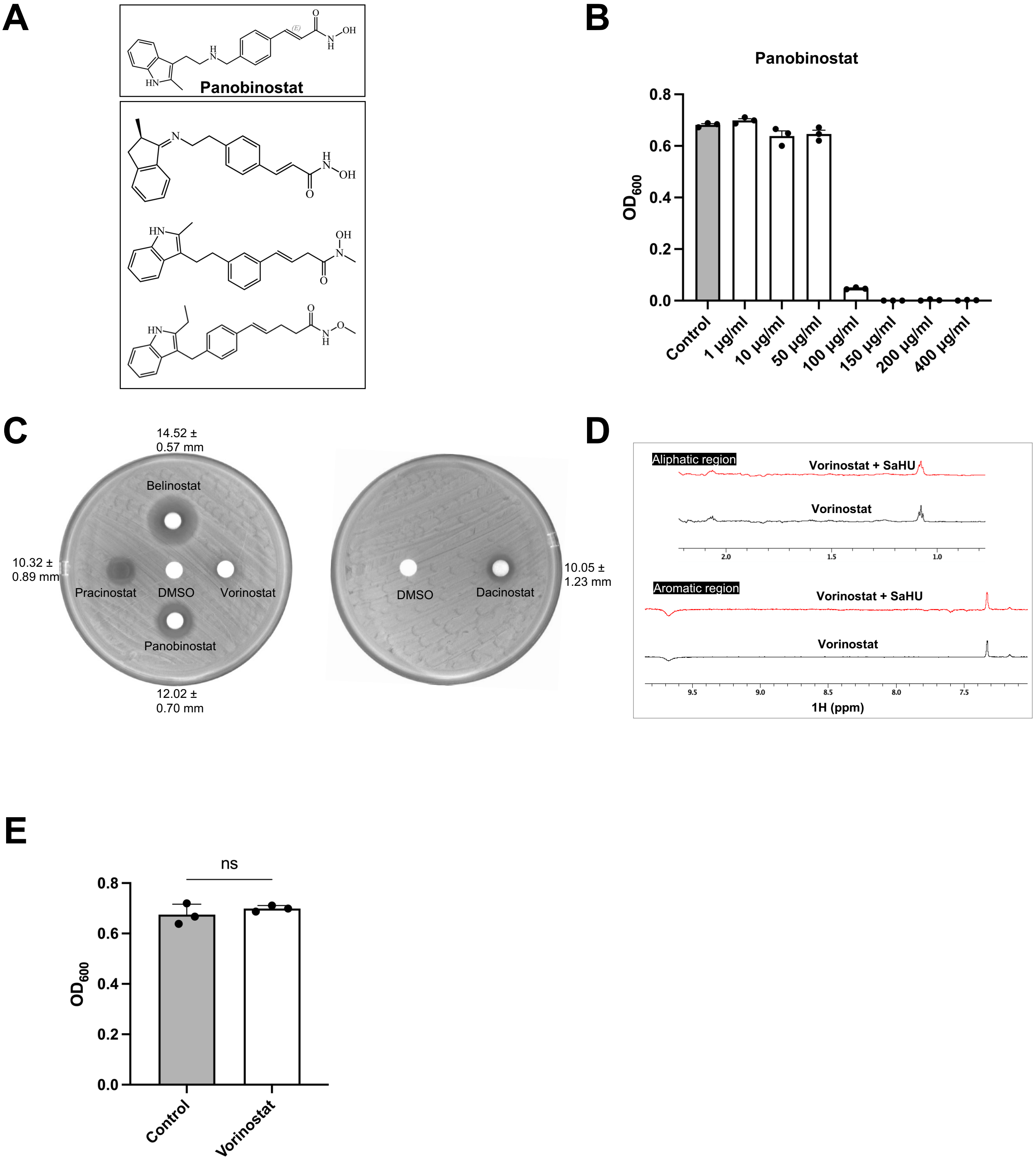

Supplementary figure 3: Lead compound library expansion and verification among HDAC inhibitors.

(**A**) The structural formulas of Panobinostat and top three optimized molecules from PGMO. (**B**) Calculation of MIC for Panobinostat on *S. aureus*. Plots show three technical replicates (n = 3), and error bars represent the SEM. **(C)** Antimicrobial susceptibility testing (AST) of Belinostat, Panobinostat, Vorinostat, Pracinostat, and Dacinostat against *S. aureus*. Zone of inhibition assay showing the diameters of the inhibition zones. 200 μg of each drug dissolved in 20 μL DMSO was loaded on each plate. Data represent three technical replicates. (**D**) 1D NMR WaterLOGSY spectra show no interaction between Vorinostat and SaHU. (**E**) Vorinostat shows no inhibitory effect at 660 μg/mL (2.5 mM) for *S. aureus.* Statistical significance determined by an unpaired t test. Plots show three technical replicates (n = 3), error bars represent the SEM, and ns denotes not significant compared to control.

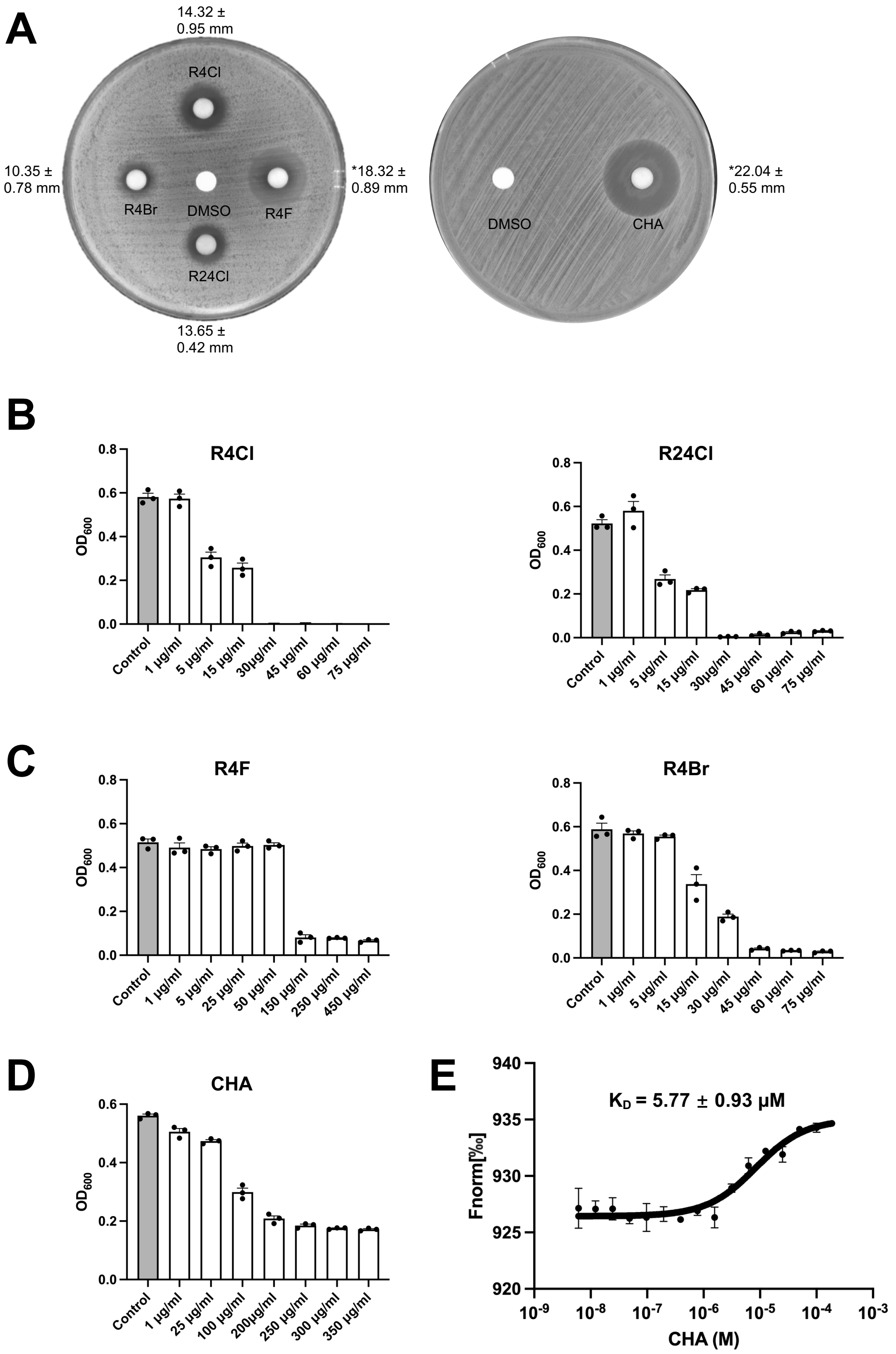

Supplementary figure 4: Lead compound library expansion and verification among CHADs. (**A**) ASTs for R4Cl, R24Cl R4F, R4Br and CHA against *S. aureus*: Zone of inhibition assay showing the diameters of the inhibition zones. 200 µg of each drug dissolved in 20 µL DMSO was loaded on each plate. Data represent three technical replicates. * denotes incomplete zone clearance for CHA and R4F. (**B**) The MIC determinations for R4Cl and R24Cl on *S. aureus.* Plots show three technical replicates (n = 3), and error bars represent the SEM. (**C**) The MIC determinations for R4F and R4Br on *S. aureus.* Plots show three technical replicates (n = 3), and error bars represent the SEM. (**D**) The MIC determination for CHA on *S. aureus.* Plots show three technical replicates (n = 3), and error bars represent the SEM. (**E**) The binding affinity between CHA and SaHU determined by MST. Plots show means, and error bars represent the SEM.

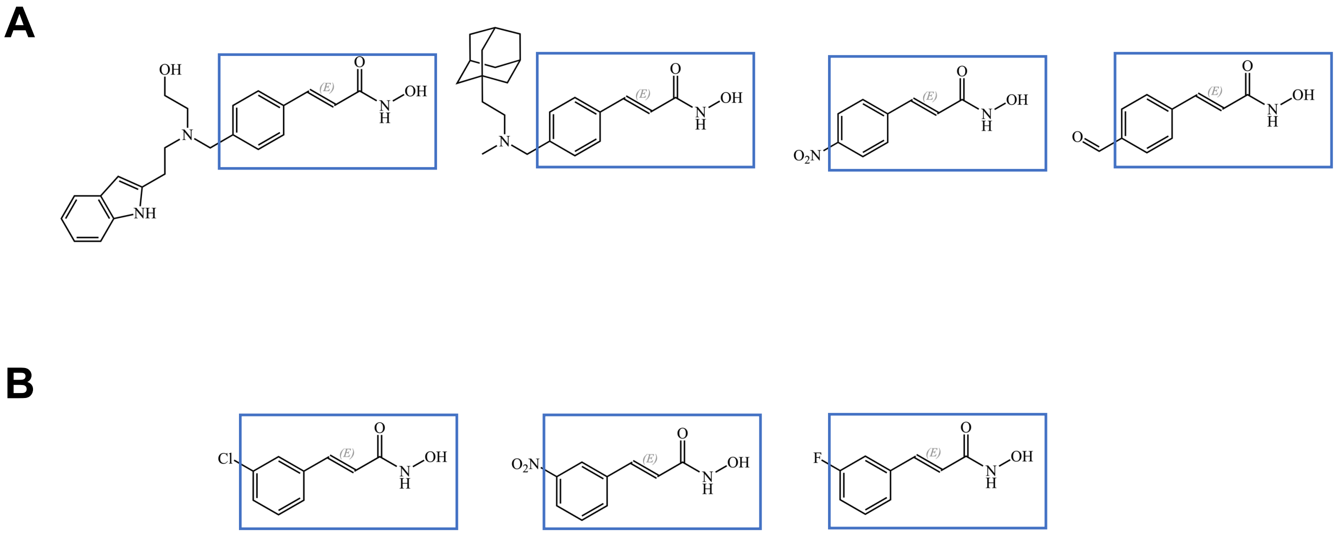

Supplementary figure 5: Additional CHADs with possible antibacterial properties.

(**A**) Additional small molecules from ZINC20 with substitutions on R4 group of CHA.

(**B**) Additional small molecules from ZINC20 with substitutions on R3 group of CHA.

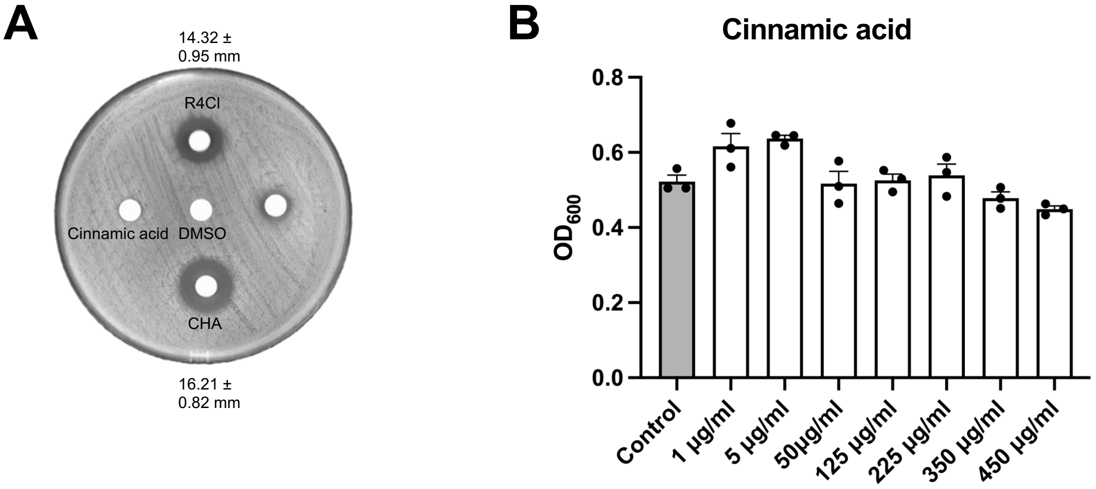

Supplementary figure 6: Cinnamic acid has no antibacterial activity against *S. aureus*.

(**A**) AST for cinnamic acid in comparison with CHA and R4Cl: Zone of inhibition assay showing the diameters of the inhibition zones. 200 µg of each drug dissolved in 20 µL DMSO was loaded on each plate. Data represent three technical replicates. (**B**) The MIC determination for cinnamic acid on *S. aureus*. Plots show three technical replicates (n = 3), and error bars represent the SEM.

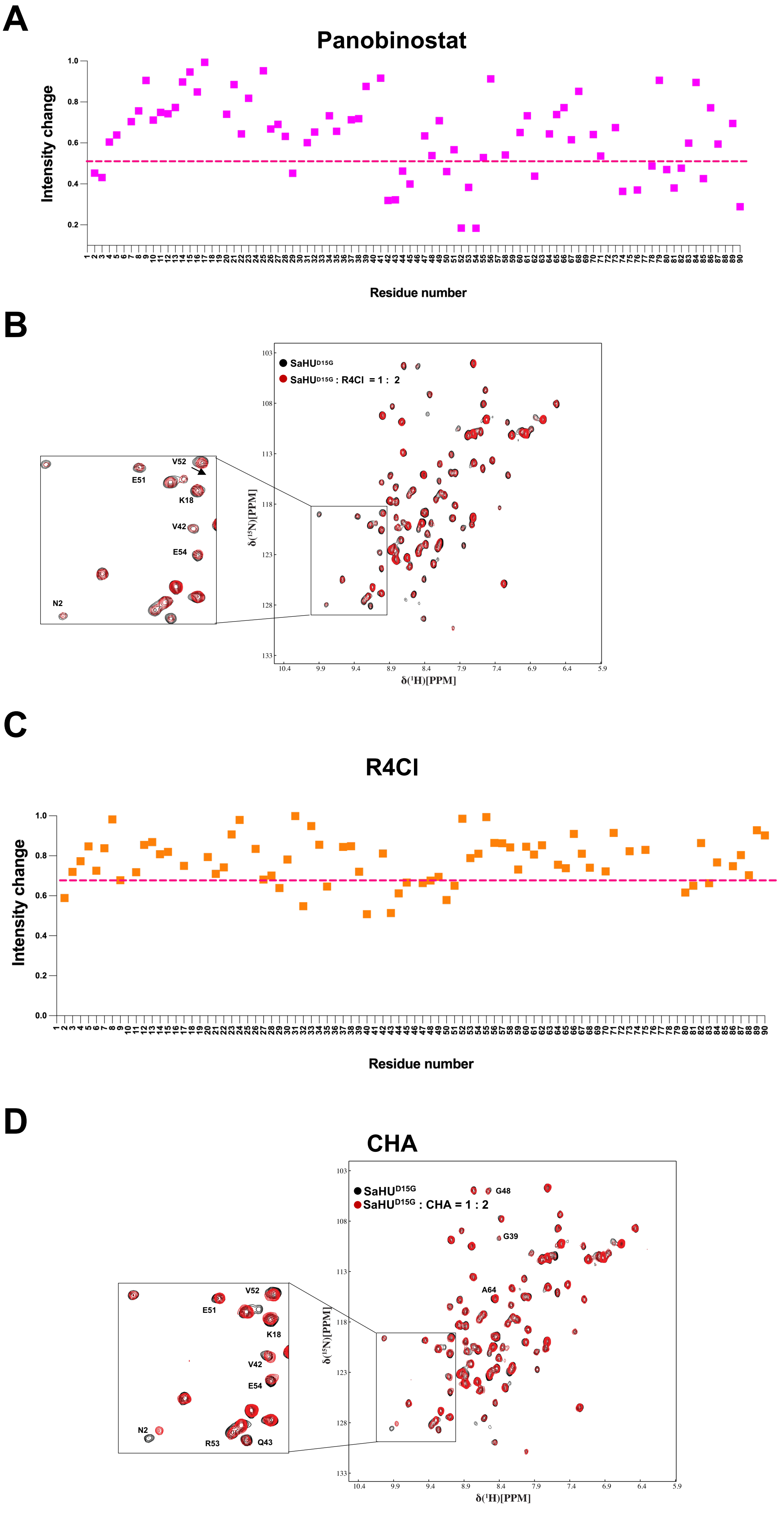

Supplementary figure 7: NMR studies of SaHU-CHADs interactions.

(A) Peak intensity changes for each residue after adding Panobinostat (dash line indicates 50% mark). **(B)** Overlay of ^{1}H-^{15}N HSQC spectra of SaHU^{D15G} (black) and SaHU^{D15G} with 5 molar equivalents of R4Cl added (red). **(C)** Peak intensity changes for each residue after adding R4Cl (dash line indicates 70% mark). (**D)** Overlay of ^{1}H-^{15}N HSQC spectra of SaHU^{D15G} (black) and SaHU^{D15G} with 5 molar equivalents of CHA added (red). The similar set of residues experienced chemical perturbations or peak broadening as seen in Panobinostat titration.

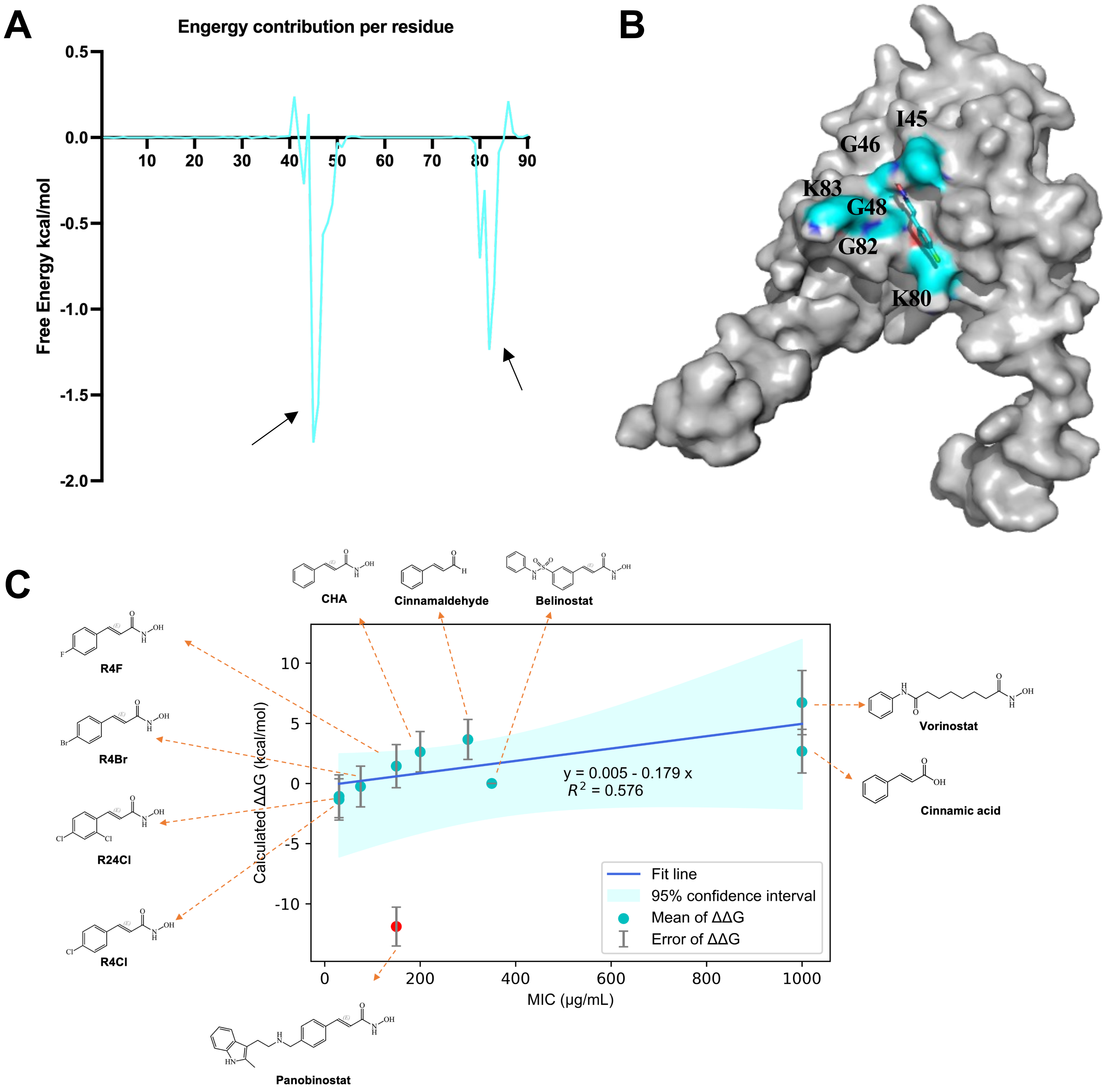

Supplementary figure 8: Free energy calculation and RBFE.

(A) The total energy contribution per residue. The arrows point to the two valleys in which the corresponding residues contribute most to the binding free energy. **(B)** Two valleys are highlighted on the structure of SaHU^{D15G} with corresponding residues labeled. **(C)** The linear correlation between calculated RBFEs (ΔΔG) and the experimentally determined MICs with Panobinostat as the only outlier.

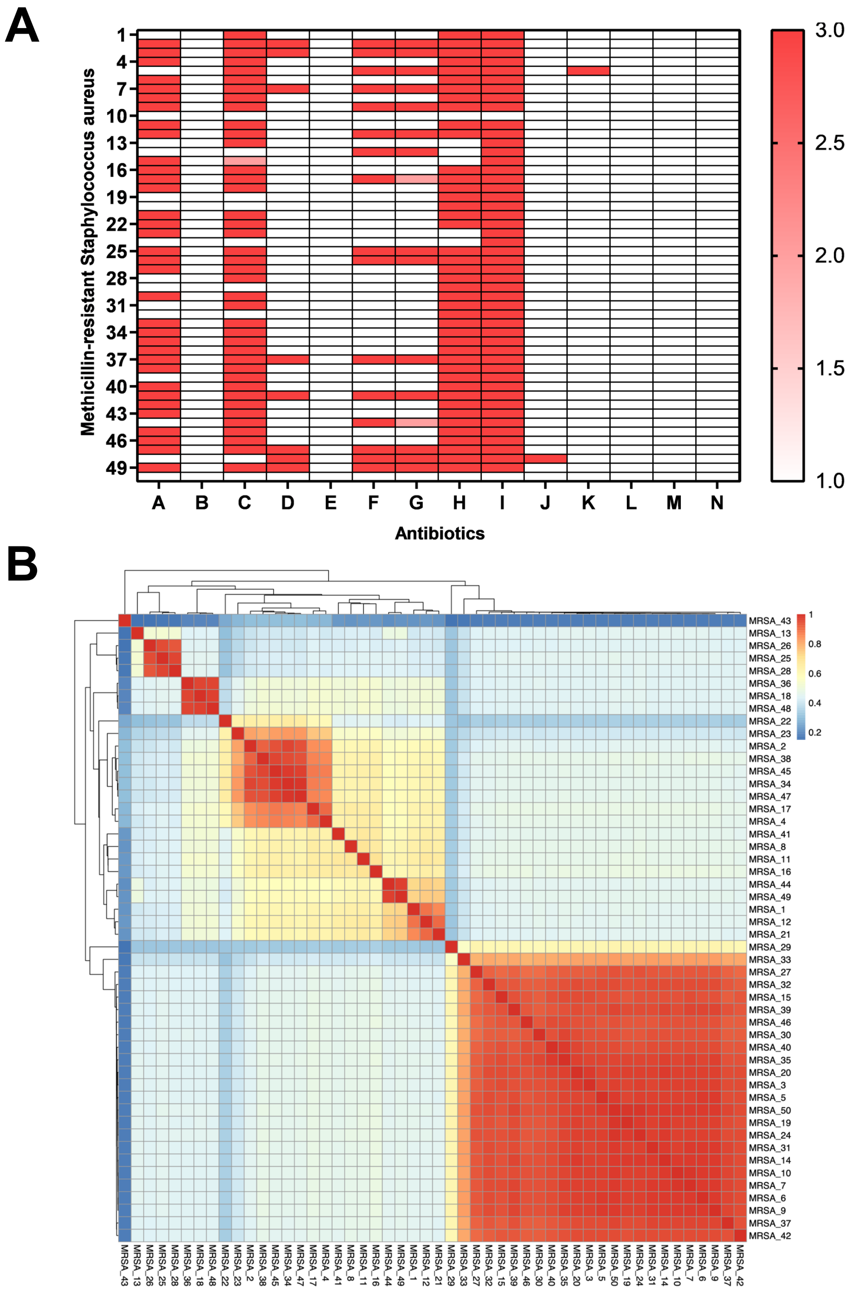

Supplementary figure 9: Antibiotic-resistant and genomic sequence profiles of 50 MRSA strains.

(A) The antibiotic resistant profiles of clinically isolated MRSA: A-Clindamycin, B-Daptomycin, C-Erythromycin, D- Gentamicin, E-Linezolid, F-Levofloxacin, G-Moxifloxacin, H-Oxacillin, I-Penicillin, J-Rifampin, K-Sulfamethoxazole, L-Teicoplanin, M-Tigecycline, N-Vancomycin. Bar: 1.0-Sensitive, 2.0-Intermediate and 3.0-Resistant. **(B)** Heatmap and dendrogram established from 50 genomes of MRSA using Sourmash “signatures”. Full matrix with rows and columns clustering 50 MRSA strains organized by their sequence similarity and MRSA-43 representing an outgroup.

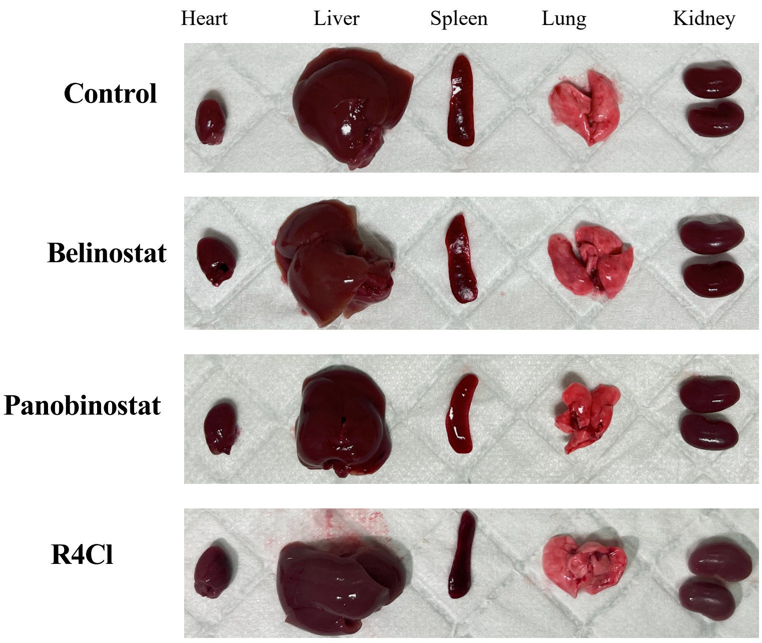

Supplementary figure 10: Toxicity of Belinostat, Panobinostat and R4Cl in mice via superficial application.

Gross autopsy of heart, liver, spleen, lungs, and kidneys.

A

Belinostat

Seq.	Dosage (mg/kg)	Short-term outcome	Long-term outcome	Symptoms
1	175	O	O	Shortness of breath, weakness, recovered after 1 hours, be dissected 14 days later
2	280	X	X	Severe tremor, weakness, dead after 5 min
3	175	O	O	Weakness, recovered after 2 hours, be dissected 14 days later
4	280	X	X	Severe tremor, weakness, dead after 5 min
5	175	O	O	Tremor, weakness, recovered after 2 hours, be dissected 14 days later

(X = Died, O = Survived)
Stopping criteria met: LR criterion
Estimated LD50 = 242.7 (Based on an assumed sigma of 0.2)
Approximate 95% confidence interval is 175 to 280.

B

Panobinostat

Seq.	Dosage (mg/kg)	Short-term outcome	Long-term outcome	Symptoms
1	175	X	X	Tremor, weakness, dead after 5 min
2	110	X	X	Tremor, weakness, dead after 5 min
3	70	O	O	Tremor, weakness, recovered after 2 hours, be dissected 14 days later
4	110	X	X	Tremor, weakness, dead after 5 min
5	70	O	O	Shortness of breath, weakness, recovered after 1 hours, be dissected 14 days later
6	110	X	X	Tremor, weakness, recovered after 2 hours

(X = Died, O = Survived)
Stopping criteria met: LR criterion
Estimated LD50 = 78.64 (Based on an assumed sigma of 0.2)
Approximate 95% confidence interval is 70 to 110.

C

R4Cl

Seq.	Dosage (mg/kg)	Short-term outcome	Long-term outcome	Symptoms
1	175	O	O	Shortness of breath, weakness, recovered after 4 hours, be dissected 14 days later
2	280	X	X	Tremor, weakness, dead after 5 min
3	175	O	O	Weakness, recovered after 2 h, be dissected 14 days later
4	280	X	X	Tremor, weakness, dead after 5 min
5	175	O	O	Weakness, recovered after 2 h, be dissected 14 days later

(X = Died, O = Survived)
Stopping criteria met: LR criterion
Estimated LD50 = 242.7 (Based on an assumed sigma of 0.2)
Approximate 95% confidence interval is 175 to 280.

Supplementary figure 11: The acute toxicity tests for intravenous injection of Belinostat, Panobinostat and R4Cl.

LD_{50} for Belinostat **(A)**, Panobinostat **(B)** and R4Cl **(C)** were determined using the up-and-down procedure.

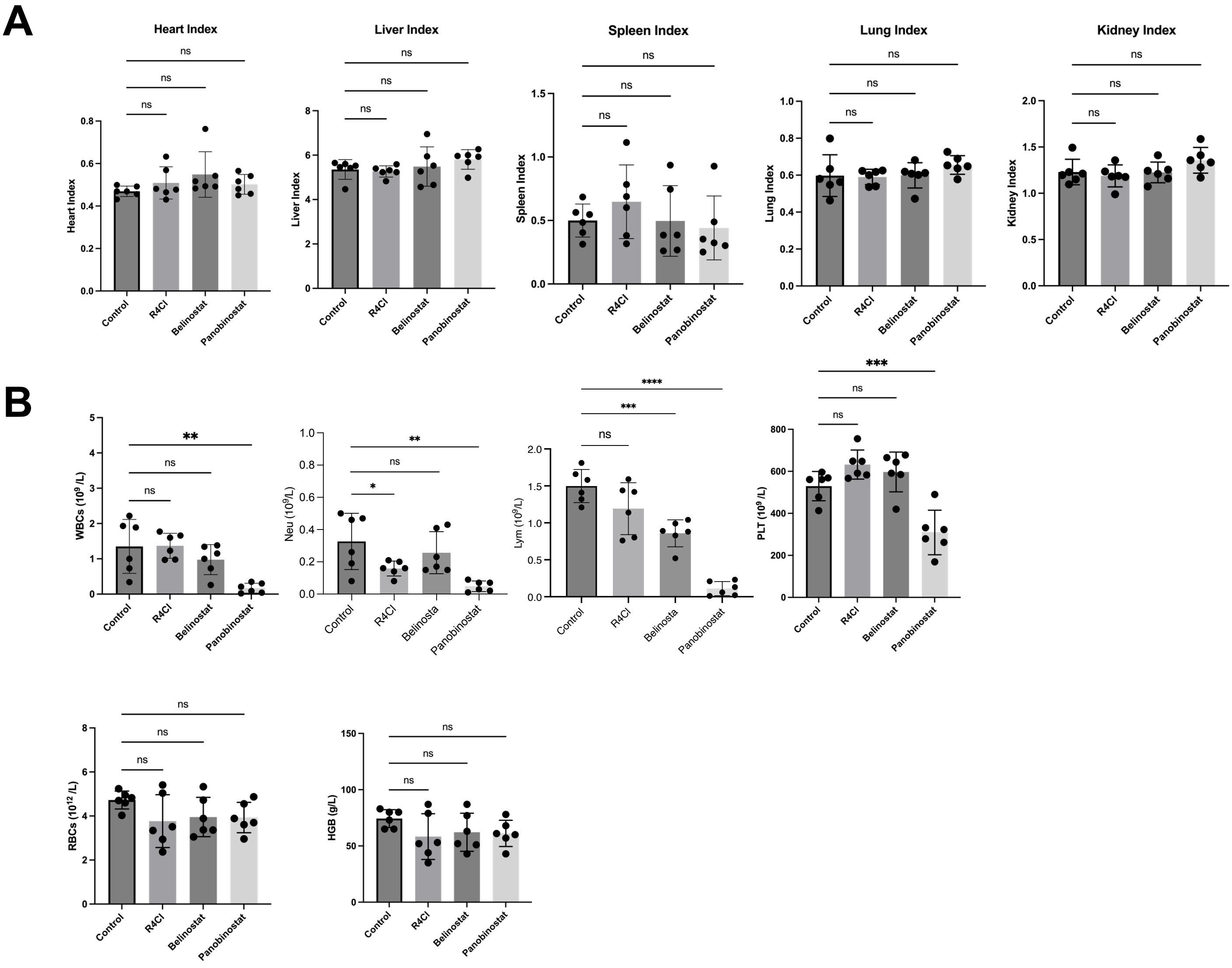

Supplementary figure 12: The 7 days toxicity tests for intravenous injection of Belinostat, Panobinostat and R4Cl in mice.

(A) The heart, liver, spleen, lung and kidney indices. Organ index (mg/g) = organ weight (mg)/body weight of mice (g). Statistical significance determined by one-way ANOVA. Plots show the individual values for each mouse (n = 6), error bars represent the SEM, and ns denotes not significant compared to control.

(B) Cell counts of WBCs, Neu, Lym, PLT, RBCs, and HGB in different groups. Statistical significance determined by one-way ANOVA. Plots show the individual values for each mouse (n = 6), and error bars represent the SEM. $^{**}P < 0.01$, $^{***}P < 0.001$, $^{****}P < 0.0001$, and ns denotes not significant compared to control.

A

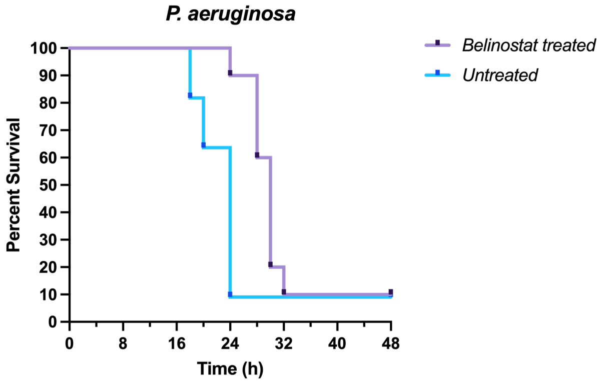

B

**Bacteria**	**MIC (μg/mL)**		
	**Tobramycin**	**Erythromycin**	**Rifamycin**
B. subtilis (168)	0.5	-	-
S. aureus (ATCC 29213)	0.5	-	-
A. baumannii (BBA-1605)	-	6	-
E. coli (MG1655)	-	4	-
K. pneumonia (ATCC 13883)	-	4	-
P. aeruginosa (PAO1)	-	1	-
M. Tuberculosis (H37Rv)	-	-	0.25

Supplementary figure 13: Survival curves and quality controls.

(A) Kaplan-Meier survival curves (n = 10) of the solvent used in this study (untreated) and Belinostat (treated) injected *P. aeruginosa* sepsis mice that monitored for 48 hours. **(B)** The quality controls of the bacteria used in the study: tobramycin for Gram-positive bacteria (except for *M. tuberculosis*), erythromycin for Gram-negative bacteria, and Rifamycin for *M. tuberculosis.*

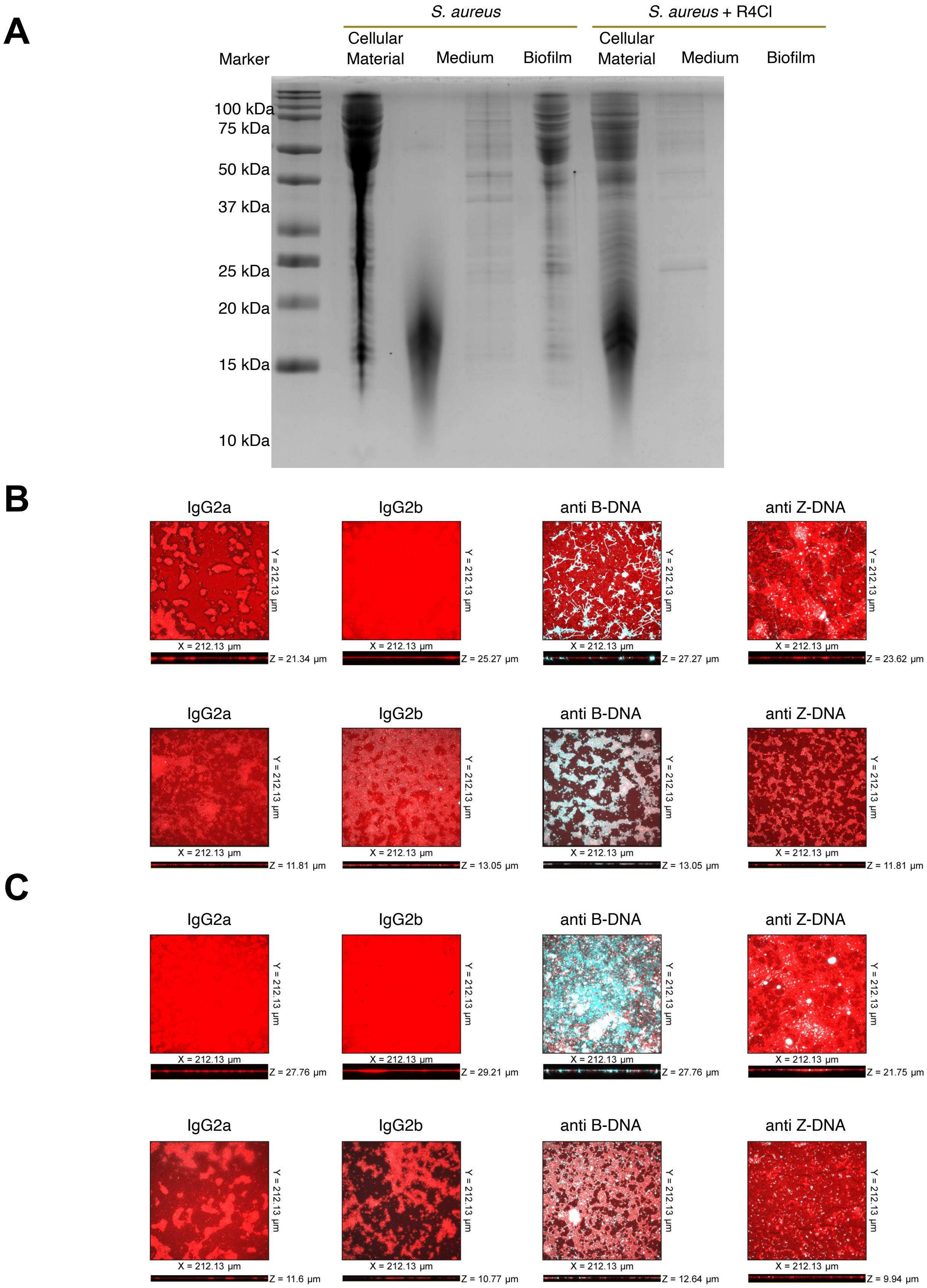

Supplementary figure 14: The effect of R4Cl on biofilm.

(A) Gel image showing the protein content of different fractions of *S. aureus* with or without R4Cl treatment. **(B)** Representative images of *P. aeruginosa* biofilms treated with DMSO (upper panels) or R4Cl (lower panels). Biofilms were incubated with antibody controls (murine isotype IgG2a or IgG2b), a murine monoclonal antibody against B-DNA, or a murine monoclonal antibody against Z-DNA, followed by goat anti-mouse IgG conjugated to Alexa Fluor 405 and FM 4-64 staining. XY images show the maximum fluorescence intensity of Z stacks, and XZ projections were generated and analyzed using ImageJ. **(C)** Representative images of *S. aureus* biofilms treated with DMSO (upper panels) or R4Cl (lower panels). Biofilms were processed identically to panel (B). XY images show the maximum fluorescence intensity of Z stacks, and XZ projections were generated and analyzed using ImageJ.

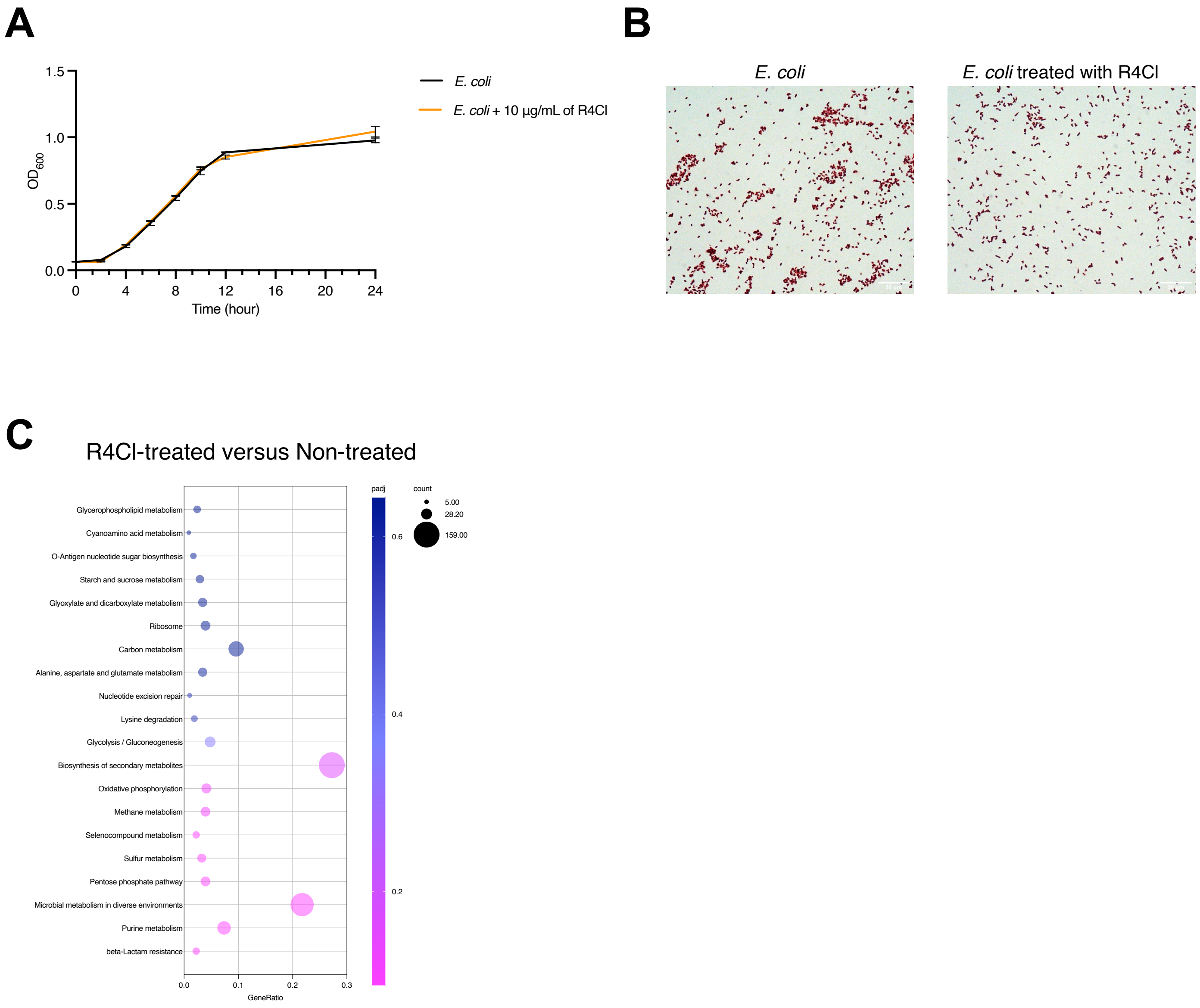

Supplementary figure 15: The effect of R4Cl on *E. coli*

(A) Growth curve of *E. coli* and *E. coli* treated with 10 µg/mL of R4Cl over 24 hours. Three biological replicates were performed, each containing three technical replicates. The growth curve is a representative experiment. Error bars represent the SEM. **(B)** The Gram staining images showing no difference between *E. coli* and *E. coli* treated with 10 µg/mL of R4Cl. Scale bar: 20 µm. **(C)** KEGG pathway enrichment analysis of differentially expressed genes between 10 µg/mL of R4Cl treated and untreated *E. coli* cells. Y-axis indicates the pathway name, x-axis indicates generatio. The bubble size indicates the number of genes and the color bar indicates the adjusted p-value.

Supplementary Video 1: Molecular dynamics simulation for the SaHU^{D15G}-R4Cl complex
