# Supplementary material for: Cinnamic‐Hydroxamic‐Acid Derivatives Exhibit Antibiotic, Anti‐Biofilm, and Supercoiling Relaxation Properties by Targeting Bacterial Nucleoid‐Associated Protein HU
Source: Adv Sci (Weinh). 2025 Nov 21;13(13):e09876. doi: 10.1002/advs.202509876 (PMC12955902; doi:10.1002/advs.202509876)
Supplement: Supplementary file 3 — Supplemental Data [file ADVS-13-e09876-s001.zip › advs72933-sup-0011-Supplementary Table 10.docx]

**Supplementary table 10**: List of abbreviations used in the study.

| **Abbreviation** | **Full Term** |
| --- | --- |
| AMR | Antimicrobial resistance |
| AST | Antibiotic susceptibility testing |
| BDF | Bisphenol derivative of fluorene |
| bTMP | Biotinylated trimethyl psoralen |
| CB | Carbenicillin |
| CHA | Cinnamic hydroxamic acid |
| CHAD | Cinnamic hydroxamic acid derivative |
| CLSI | Clinical and Laboratory Standards Institute |
| CPI | Compound protein interaction |
| dsDNA | Double-stranded DNA |
| eDNA | Extracellular DNA |
| EPS | Extracellular polymeric substances |
| EOP | Efficiency of plating |
| ESKAPE  EMSA | *Enterococcus faecium*, *Staphylococcus aureus*, *Klebsiella pneumoniae*, *Acinetobacter baumannii*, *Pseudomonas aeruginosa*, and *Enterobacter* spp.  Electrophoretic Mobility Shift Assay |
| FA | Fusidic acid |
| FIC | Fractional inhibitory concentration |
| FoR | Frequency of resistance |
| Gp46 | Gene product 46 (phage SPO1 protein) |
| HA | Hydroxamic acid |
| HADDOCK | High Ambiguity Driven protein–protein Docking |
| H&E | Hematoxylin-eosin |
| HDACi | Histone deacetylase inhibitor |
| HGB | Hemoglobin |
| HSQC | Heteronuclear single quantum coherence |
| HU | Histone-like protein HU (from *Escherichia coli* strain U93) |
| IHF | Integration host factor |
| KD | Dissociation constant |
| LPS | Lipopolysaccharide |
| MIC | Minimum inhibitory concentration |
| MD | Molecular dynamics |
| MRSA | Methicillin-resistant *Staphylococcus aureus* |
| MST | Microscale thermophoresis |
| NAP | Nucleoid-associated protein |
| Neu | Neutrophils |
| NMR | Nuclear magnetic resonance |
| PDX | Patient-derived xenograft |
| PGMO | Pangu Molecule Optimizer |
| PLT | Platelets |
| RBFE | Relative binding free energy |
| RBC | Red blood cells |
| R4Cl | CHAD molecule with chlorine substitution at R4 position |
| R4Br | CHAD molecule with bromine substitution at R4 position |
| R4F | CHAD molecule with fluorine substitution at R4 position |
| SaHU | *Staphylococcus aureus* HU protein |
| SEM | Scanning electron microscopy |
| SD | Stilbene derivative |
| TEM | Transmission electron microscopy |
| WBC | White blood cells |
| WaterLOGSY | Water Ligand Observed Gradient Spectroscopy |
| XDR-TB | Extensively drug-resistant tuberculosis |
| Z-DNA | Left-handed helical form of DNA |
