# Supplementary material for: Cinnamic‐Hydroxamic‐Acid Derivatives Exhibit Antibiotic, Anti‐Biofilm, and Supercoiling Relaxation Properties by Targeting Bacterial Nucleoid‐Associated Protein HU
Source: Adv Sci (Weinh). 2025 Nov 21;13(13):e09876. doi: 10.1002/advs.202509876 (PMC12955902; doi:10.1002/advs.202509876)
Supplement: Supplementary file 3 — Supplemental Data [file ADVS-13-e09876-s001.zip › advs72933-sup-0008-Supplementary Table 7.pdf]

Supplementary Table 5. Primers used in Cloning and Mutagenesis

***Staphylococcus aureus* HU**		
Plasmid	pET SUMO-SaHU	
Primer	Vector Linearization	Sense: ACCACCAATCTGTTC
		Antisense: TAATAGCTCGAGCAC
	SaHU Amplification	Sense: GAACAGATTGGTGGTATGAACAAAACAGATTTAATC
		Antisense: GTGGTGCTCGAGCTATTATTTACAGCATCTTTTAATG
Plasmid	pET SUMO-SaHU^{D15G}	
Primer	SaHU^{D15G}	Sense: AGAGCAAGCTGGTTTAACTAAAAAAG
		Antisense: GCAACTGCATTGATTAAATC
Plasmid	pET28a-SaHU-6His	
Primer	Vector Linearization	Sense: GGTATATCTCCTTCTTAAAG
		Antisense: GGAGCAGGGGC
	SaHU Amplification	Sense: GAAGGAGATATACCATGAACAAAACAGATTTAATC
		Antisense: GCAGCCGGATCTCATTAGTGGTGGTGGTGGTGGTGTTTT ACAGCATCTTTTAATG
Plasmid	pET28a-SaHU^{D15G}-6His	
Primer	SaHU^{D15G}	Sense: AGAGCAAGCTGGTTTAACTAAAAAAG
		Antisense: GCAACTGCATTGATTAAATC
***Bacillus subtilis* HU**		
Plasmid	pET28a-HBsu-6His	
Primer	HBsu Amplification	Sense: GAAGGAGATATACCATGAACAAAACGGAGCTC
		Antisense: GCAGCCGGATCTCATTAGTGGTGGTGGTGGTGGTGTTTG CCGGCAACG
***Mycobacterium tuberculosis* HU**		
Plasmid	pET28a-Mtb_HU(1-94)-6His	
Primer	Mtb_HU Amplification	Sense: GAAGGAGATATACCATGAACAAAGCAGAGC
		Antisense: GCAGCCGGATCTCAACGCTGCGCG

***Streptococcus pneumoniae* HU**		
Plasmid	pET28a-Sn_HU	
Primer	Sn_HU Amplification	Sense: GAAGGAGATATACCATGGCAAACAAACAAGATTTG
		Antisense: GCAGCCGGATCTCATTAGTGGTGGTGGTGGTGGTGTTT AACAGCGTCTTTAAGAGC
***Streptococcus pyogenes* HU**		
Plasmid	pET28a-Sp_HU	
Primer	Sp_HU Amplification	Sense: GAACAGATTGGTGGTATGAACAAGACTCAACTG
		Antisense: GTGGTGCTCGAGCTATTACTTAACTGCGTCT TTCAG
***Acinetobacter baumannii* HU**		
Plasmid	pET28a-Ab_HU-6His	
Primer	Ab_HU Amplification	Sense: GAAGGAGATATACCATGAATAAATCAGAATTAATCGATGC
		Antisense: GCAGCCGGATCTCATTAGTGGTGGTGGTGGTGGTGAGC AACTGAATCTTTAAGACC
**dsDNA**		
DNA1		TGCTTATCAATTTGTTGCACC
DNA2		GGTGCAACAAATTGATAAGCA
