# Supplementary material for: Cinnamic‐Hydroxamic‐Acid Derivatives Exhibit Antibiotic, Anti‐Biofilm, and Supercoiling Relaxation Properties by Targeting Bacterial Nucleoid‐Associated Protein HU
Source: Adv Sci (Weinh). 2025 Nov 21;13(13):e09876. doi: 10.1002/advs.202509876 (PMC12955902; doi:10.1002/advs.202509876)
Supplement: Supplementary file 3 — Supplemental Data [file ADVS-13-e09876-s001.zip › advs72933-sup-0009-Supplementary Table 8.docx]

**Supplementary table 8:** X-ray data collection and processing statistics

|  | **Sa_Hu** |
| --- | --- |
| Space group | *P*12_1_1 |
| Unit cell (Å) | *a* = 41.427,  *b* = 82.369,  *c* = 61.633 |
| Wavelength (Å) | 0.9789 |
| Resolution^a^ (Å) | 29.19–2.143 (2.22–2.143) |
| Unique reflections | 22347 |
| Completeness^a^ (%) | 97.95 (97.29) |
| Intensity (*I*/σ) ^a^ | 12.62 (2.32) |
| Wilson B-factor | 40.19 |
| *R*_merge_^a^ (%) | 8.0(98.3) |
| *R*_work_ (%) | 20.81 |
| *R*_free_ (%) | 24.57 |
| Average B factors (A^2^) | 50.7 |
| No. of protein residues | 360 |
| No. of water | 93 |
| RMSD of bond length (Å) | 0.008 |
| RMSD of bond angle (°) | 0.88 |

^a^Values in parentheses refer to the last shell.
